# Molecular and serological surveys of canine distemper virus: A meta-analysis of cross-sectional studies

**DOI:** 10.1371/journal.pone.0217594

**Published:** 2019-05-29

**Authors:** Vivaldo Gomes da Costa, Marielena Vogel Saivish, Roger Luiz Rodrigues, Rebeca Francielle de Lima Silva, Marcos Lázaro Moreli, Ricardo Henrique Krüger

**Affiliations:** 1 Enzymology Laboratory, Department of Cell Biology, University of Brasilia, Brasília, Federal District, Brazil; 2 Virology Laboratory, Institute of Health Sciences, Federal University of Goiás, Jataí, Goiás, Brazil; Faculty of Science, Ain Shams University (ASU), EGYPT

## Abstract

**Background:**

Canine morbillivirus (canine distemper virus, CDV) persists as a serious threat to the health of domestic dogs and wildlife. Although studies have been conducted on the frequency and risk factors associated with CDV infection, there are no comprehensive data on the current epidemiological magnitude in the domestic dog population at regional and national levels. Therefore, we conducted a cross-sectional study and included our results in a meta-analysis to summarize and combine available data on the frequency and potential risk factors associated with CDV infection.

**Methods:**

For the cross-sectional study, biological samples from dogs suspected to have canine distemper (CD) were collected and screened for viral RNA. Briefly, the PRISMA protocol was used for the meta-analysis, and data analyses were performed using STATA IC 13.1 software.

**Results:**

CDV RNA was detected in 34% (48/141) of dogs suspected to have CD. Following our meta-analysis, 53 studies were selected for a total of 11,527 dogs. Overall, the pooled frequency of CDV positivity based on molecular and serological results were 33% (95% CI: 23–43) and 46% (95% CI: 36–57), respectively. The pooled subgroup analyses of clinical signs, types of biological samples, diagnostic methods and dog lifestyle had a wide range of CDV positivity (range 8–75%). Free-ranging dogs (OR: 1.44, 95% CI: 1.05–1.97), dogs >24 months old (OR: 1.83, 95% CI: 1.1–3) and unvaccinated dogs (OR: 2.92, 95% CI: 1.26–6.77) were found to be positively associated with CDV infection. In contrast, dogs <12 months old (OR: 0.36, 95% CI: 0.20–0.64) and dogs with a complete anti-CDV vaccination (OR: 0.18, 95% CI: 0.05–0.59) had a negative association.

**Conclusion:**

Considering the high frequency of CDV positivity associated with almost all the variables analyzed in dogs, it is necessary to immediately and continuously plan mitigation strategies to reduce the CDV prevalence, especially in determined endemic localities.

## Introduction

Canine morbillivirus (previously known as canine distemper virus (CDV)) is one of the major pathogens in canine populations, as it causes one of the most contagious and fatal diseases for domestic dogs (*Canis familiaris*) [1,2]. CDV is enveloped with single-stranded, negative sense and nonsegmented RNA genetic material, belonging to the genus *Morbillivirus* (family *Paramyxoviridae*) [3]. Viral transmission occurs via aerosols or by direct contact of susceptible animals with the various fresh body secretions of infected animals [4]. Consequently, CDV infection results in canine distemper (CD), which is a severe disease with multisystemic clinical signs [5]. Despite the existence of a vaccine, several reports highlight CDV, calling attention to the increased activity, genetic diversity and reemergence of other infections in the world [6–8].

Regarding the diagnosis of CD, it is essential to use laboratory tests with better accuracy for viral detection. This is due to the broad clinical spectrum of signs of the disease, making clinical diagnosis difficult since nonspecific clinical signs may be confused with several other infectious diseases [9–11]. Therefore, laboratory tests, including serological and molecular surveys, have been carried out over the past few years to describe the epidemiological profile of CDV in some localities [12,13]. In these laboratory tests, methodological variants aim at the specific identification of genetic material, antigens and proteins (IgG and/or IgM) related to CDV. For this purpose, several biological samples, including nasal, ocular and saliva secretions and blood, feces, urine and infected tissues have been used mainly for PCR, immunochromatography (IC), seroneutralization (SN), immunofluorescence (IFA) and ELISA [14–16] analyses. However, there are still several gaps related to CDV epidemiology, including the following: 1) the frequencies of infections in domestic dogs are still poorly characterized; 2) the sample size of most studies is relatively small; 3) there is no robust analysis of the risk factors associated with CD/CDV; 4) there is no synthesis of the current epidemiological picture regarding the burden of the disease and its frequency according to various clinical signs, diagnostic methods and types of biological samples analyzed.

In view of the significant impact of CDV infections on the health of domestic dogs, which are the main reservoir hosts, and the lack of data on the epidemiological characteristics of these infections in the world, this observational study and meta-analysis aimed to determine and better understand the individual and pooled frequency patterns of detectable CDV using various molecular and serological tests.

## Methods

### Cross-sectional study

With the main purpose of laboratory diagnosis of CDV, the present study was approved by the ethics committee on the use of animals of the Universidade Federal de Goiás (Protocol Number: 054/17). Samples from domestic dogs showing clinical signs suggestive of CD were collected between 2017 and 2019. The collection sites were the Veterinary Hospital of the UFG and the Control Center of Zoonoses of the municipality of Jataí, located in the Center-West region of Brazil.

After blood samples were collected in tubes (BD Vacutainer PPT 13x100 mm, 5 ml) and nasal specimens were collected with flocked swabs placed into 1 ml universal transport medium (UTM (Copan, Brescia, Italy)) for the purpose of molecular diagnostic testing, the plasma and UTM were separated and used for the detection of viral RNA. Initially, the RNA was extracted using a QIAamp Viral RNA commercial kit (Qiagen, Hilden, Germany) according to the manufacturer’s specifications. Subsequently, following adaptations of the protocol of Castilho et al. and Frisk et al. [17,18], reverse transcription, PCR and nested PCR were performed for the purpose of partial detection of the CDV nucleoprotein (N) gene. After the addition of the possible amplicons in the 1.5% agarose gel stained with SYBR Safe DNA gel stain (Invitrogen; Carlsbad, USA), the amplification product was analyzed under ultraviolet light. The molecular identity of the PCR product of expected size (287 bp) was confirmed by DNA sequencing (ACTGene Análises Moleculares Ltda., RS, Brazil).

### Systematic review and meta-analysis

The present meta-analysis followed the methods developed in the Preferred Reporting Items for Systematic reviews and Meta-Analysis (PRISMA) protocol, which refers to rules and guidelines for systematic reviews and meta-analyses (S1 File) [19]. Additionally, we recorded the study protocol in SYRCLE (Systematic Review Center for Laboratory Animal Experimentation) (www.syrcle.nl). We also deposited our laboratory protocols at protocols.io, which can be viewed at https://dx.doi.org/10.17504/protocols.io.2umgeu6.

#### Search strategy

After defining the research protocol, we performed a systematic search in the PubMed, SciELO and ScienceDirect databases. Articles in the English, Spanish and Portuguese languages that were screened from July to October 2018 were selected. At this stage, to refine the studies of interest, a combination of descriptors was used (“*canine distemper virus*,” “*canine distemper*,” “*viruses in dogs*,” “*dogs*,” “*domestic dogs*,” “*canis familiaris*,” and “*canis lupus familiaris*”). We also sought additional studies through screening the references of selected articles and highly cited reviews of the topic of interest.

The next step involved the analysis of the selected articles containing the previously mentioned descriptors. To do so, the following inclusion criteria were used: 1) original articles published in scientific journals that contained information on serological and molecular surveys for the detection of CDV in domestic dogs; 2) studies containing data related to the proportion/rate of viral infection by laboratory tests; 3) seroepidemiological surveys for the detection of anti-CDV antibodies that included data concerning groups of animals not vaccinated against CDV; 4) data secondary to CDV positivity to analyze risk factors such as gender, age, vaccine status, breed, coinfection and lifestyle (free-ranging dogs versus non-free-ranging dogs); and 5) studies that used the most conventional ante-mortem detection tests. Regarding the exclusion criteria, the following parameters were adopted: 1) absence or confusing specification of the outcome of interest regarding the CDV positivity of laboratory tests; 2) revisions, book chapters, and seroprevalence studies not involving domestic dogs; and 3) small scale studies with a sample size <50.

#### Data analysis

For all selected studies, the following data were extracted: first author, year of publication, place of study, baseline characteristics of the studies including mean age, sex percentage, dog lifestyle, method of diagnosis, number of dogs investigated for CDV infection, proportion of positive animals, and clinical sign of CD and vaccine status. The main outcomes of interest in the data analysis were: 1) the proportion of CDV cases (laboratory confirmed to clinically suspected CD-positive dogs); 2) the proportion of cases with recent and/or previous CDV infection that were laboratory confirmed among apparently healthy dogs; and 3) the proportions of positive cases compared to the types of biological samples, clinical signs, diagnostic methods and origin of the studies. The secondary outcomes represented the determination of previously cited risk factors compared to the CDV positivity. For the bias risk analysis, a modified Joanna Briggs Institute and Strengthening the Reporting of Observational Studies in Epidemiology checklist were used [20,21]. In addition, the quality assessment of the studies referred to a modified method composed of the participant selection methodology, laboratory tests and outcome variables (S2 File).

#### Statistical analyses

Data collection required for analysis of the primary and secondary outcomes were initially extracted using Microsoft Excel (S3 File). Several tables were generated containing dichotomous data (occurrence or not of an event of interest) for the relative and cumulative calculation of the frequencies of the outcomes of interest, and a 95% confidence interval (CI) was used whenever possible. For all of the meta-analysis procedures, STATA IC/64 version 13.1 software was used (Stata Corporation, College Station, TX, USA). In STATA, the metaprop, metafunnel and metaninf commands were used for data analysis and the generation of forest and funnel plots. The relative frequency was determined by the number of cases (CDV positivity) divided by the total number of animals screened, and the results were expressed as percentages. The variance of each frequency estimate (known as ES (Effect Sizes)) was calculated as pq/n, where p is the frequency, q is 1 –p, and n is the total number of animals screened [22]. 95% Confidence intervals (CI) for the average ES were calculated with the formula: 95% CI = ES ± 1.96 * SE, where SE is the standard error (SE = √(pq/n)). To ensure proportionate weight distribution to studies presenting extreme frequency (near 0 or 1), we applied the Freeman-Tukey arcsine methodology [23,24]. In addition, the dichotomous data of the selected studies were extracted and plotted in a 2x2 table to obtain individual and combined odds ratios (ORs). The I^2^ test was also used to assess the existence of heterogeneity between studies (I^2^ = 75–100%, p<0.05) [25]. Due to the nature of the studies, the existence of heterogeneity was expected; therefore, we chose to use the random effects model for the meta-analysis as proposed by DerSimonian-Laird [26]. We performed a sensitivity analysis to test the effect of the individual influence of each study on the overall estimate, and a subgroup analysis was also performed to reduce the existence of heterogeneity. In addition, we evaluated the existence of publication bias by visual inspection of Begg’s funnel plot as well as by Egger’s test calculations [27,28].

## Results

### Cross-sectional study

To diagnose, contribute to molecular surveillance and trace the epidemiological profile of CDV in the study region, 141 clinical samples were collected from dogs exhibiting signs suggestive of CD. The mean age was 39 months (range 2–204), and most dogs were females (53%). Regarding clinical signs, in addition to ocular and nasal secretions, there was a predominance of neurological complications (myoclonus, ataxia, and paralysis of the limbs) and systemic complications (apathy and prostration). Because of these complications, 76% (107/141) of dogs died; CDV RNA was detected from nasal and blood samples in 34% (48/141) of dogs. The molecular identity was obtained by sequencing the amplicons generated by nested PCR, and the sequenced amplicons were identified with 99% homology to the partial segment of the CDV N gene.

### Systematic review and meta-analysis: Characteristics of included studies

**Fig 1:**
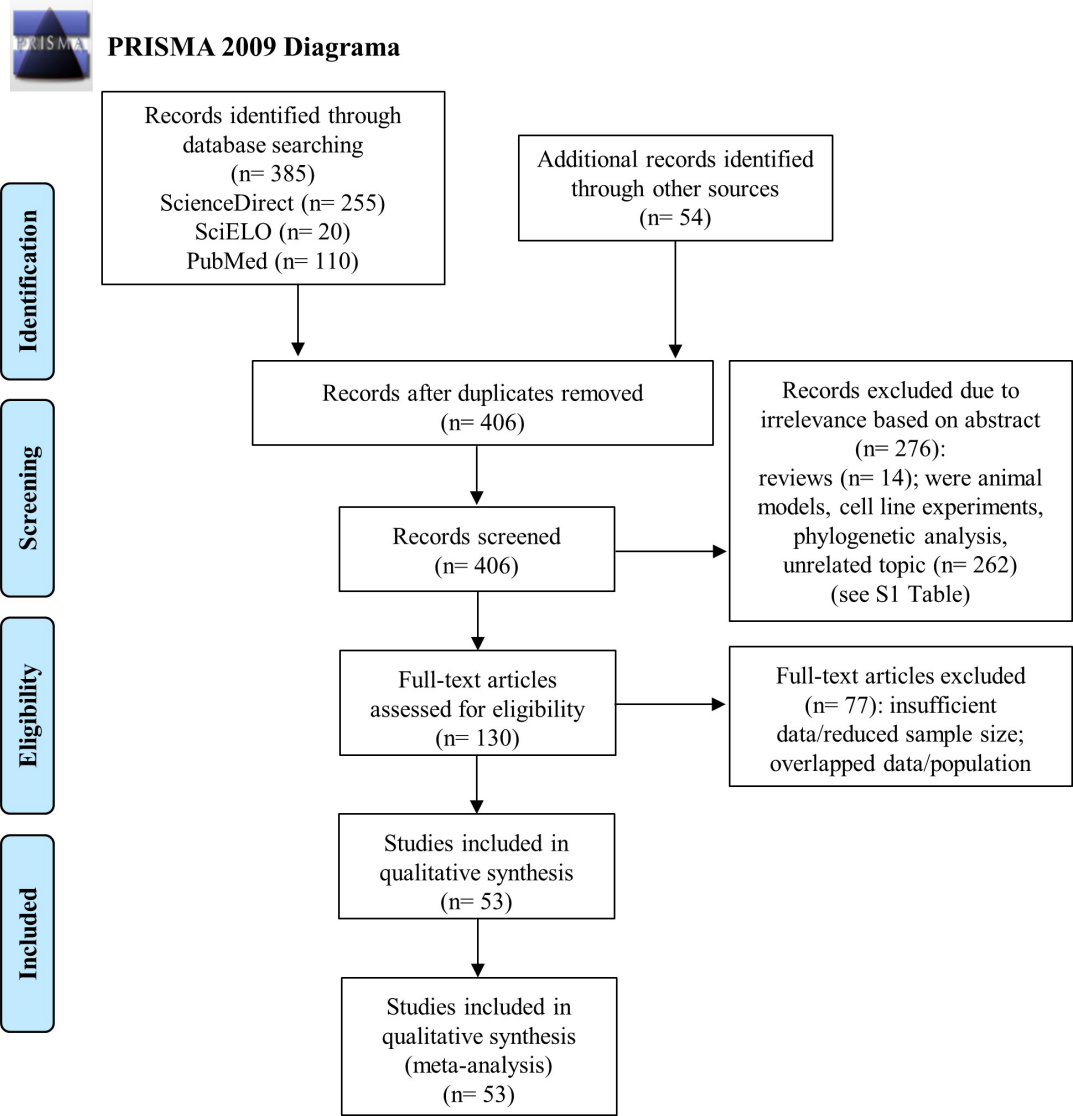
PRISMA 2009 flow diagram of observational studies included in the meta-analysis.

Initially, during the search for articles in the digital databases and additional records from other sources, 439 reference studies were found (S1 Table). After application of the inclusion and exclusion criteria, we refined the results, and 53 eligible studies constituted the present meta-analysis [14,16,29–79]. The flowchart of this selection step is shown in Fig 1.

The 53 articles selected in addition to the data from our study produced a total sample of 11,527 domestic dogs included in the CDV infection analysis (S2 Table). The age range of these animals was considerably heterogeneous, ranging from 40 days to 18 years. In some studies, the mean age was 34.5 months (±41.2) [30,42–44]. Regarding the general age profile of the animals, only a few authors specified this profile in detail. In this context, there was an approximate ratio of 2.5:1:2.4 in relation to the number of dogs included in the classification of <12 months of age, 12–24 months and >24 months of age, respectively. Thus, the majority of the animals included in the risk analysis were pups (<12 months, n = 2581) and adults (>24 months, n = 2497). In contrast to the cross-sectional study, there was a higher proportion of males than females (1.4:1).

**Fig 2:**
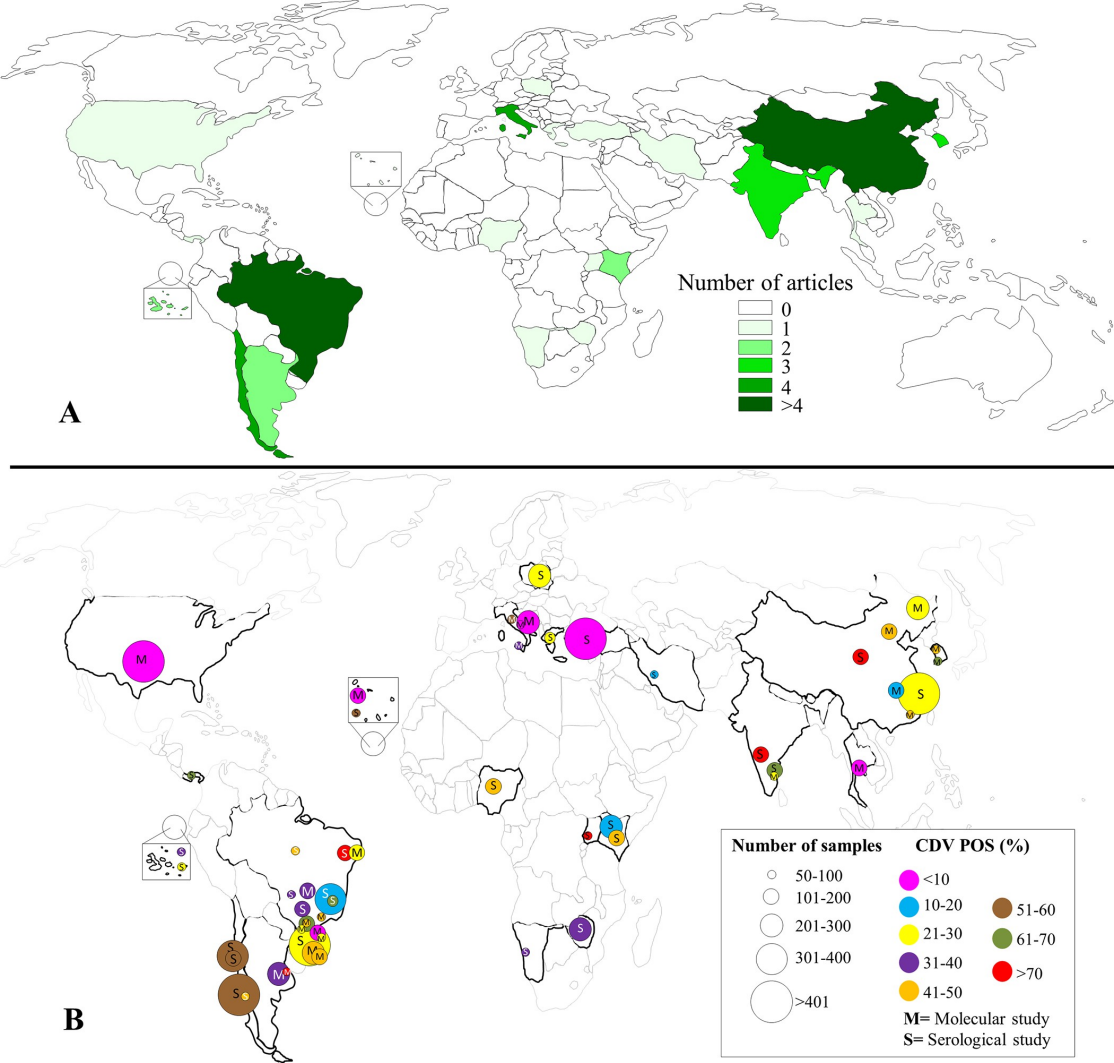
World map with the geographical distribution of the studies. The color intensity categories represent the number of studies included in the meta-analysis (A). The individual estimated frequency of laboratory confirmed CDV positivity in domestic dogs is shown (B). The CDV positivity is represented by the different colors and the total number of animals screened by the size of the circles.

Regarding the regions of origin of the selected articles, studies were conducted in 21 countries of the American (n = 6), Asian (n = 6), African (n = 6) and European (n = 3) continents. Most of the samples consisted of regions of China (n = 3104), Brazil (n = 2916) and Chile (n = 1055). More details regarding the regional distribution of CDV infection in rates are shown in Fig 2 (S3 Table).

### A meta-analysis to estimate the pooled frequency of the CDV

**Fig 3:**
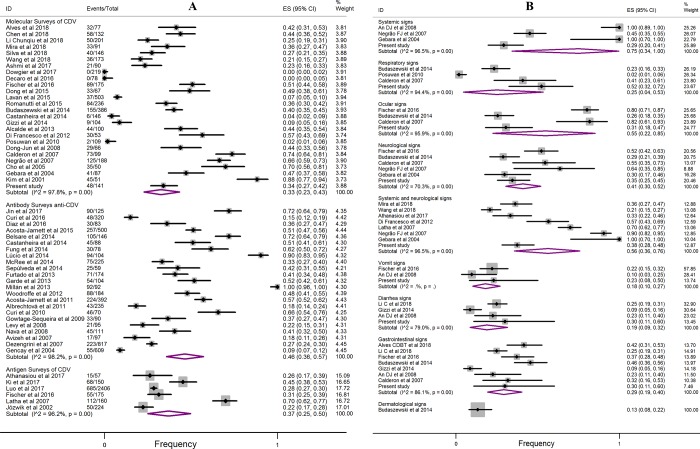
Forest plot of the frequency of laboratory confirmed CDV in biological samples from domestic dogs. Fig 3A shows the analysis of the subgroups of molecular, antibody and antigen surveys. Fig 3B shows the subgroup regarding the clinical signs of dogs (molecular surveys). The length of the line indicates the 95% confidence interval for each study, and the diamonds represent the pooled estimate. ID = identification of study; ES = effect size; Events = CDV POS.

As a result of laboratory confirmation by molecular surveys, the overall estimate of the combined frequency of CDV infection was 33% (95% CI: 23–43), with considerable evidence for regional epidemiological variations (Figs 3A and 2). For serological surveys (antibody survey anti-CDV), the pooled frequency was 46% (95% CI: 36–57), while analysis based on antigenic results was 37% (95% CI: 25–50).

To better understand the picture of current studies in relation the frequency of CDV infection versus clinical signs of animals, Fig 3B was generated. Consequently, a higher viral positivity was observed when systemic clinical signs were present (75%, 95% CI: 34–100), followed by systemic and neurological (56%, 95% CI: 36–76), ocular (55%, 95% CI: 22–85), neurological (41%, 95% CI: 30–52), gastrointestinal (29%, 95% CI: 19–40) and respiratory signs (25%, 95% CI: 4–53).

**Fig 4:**
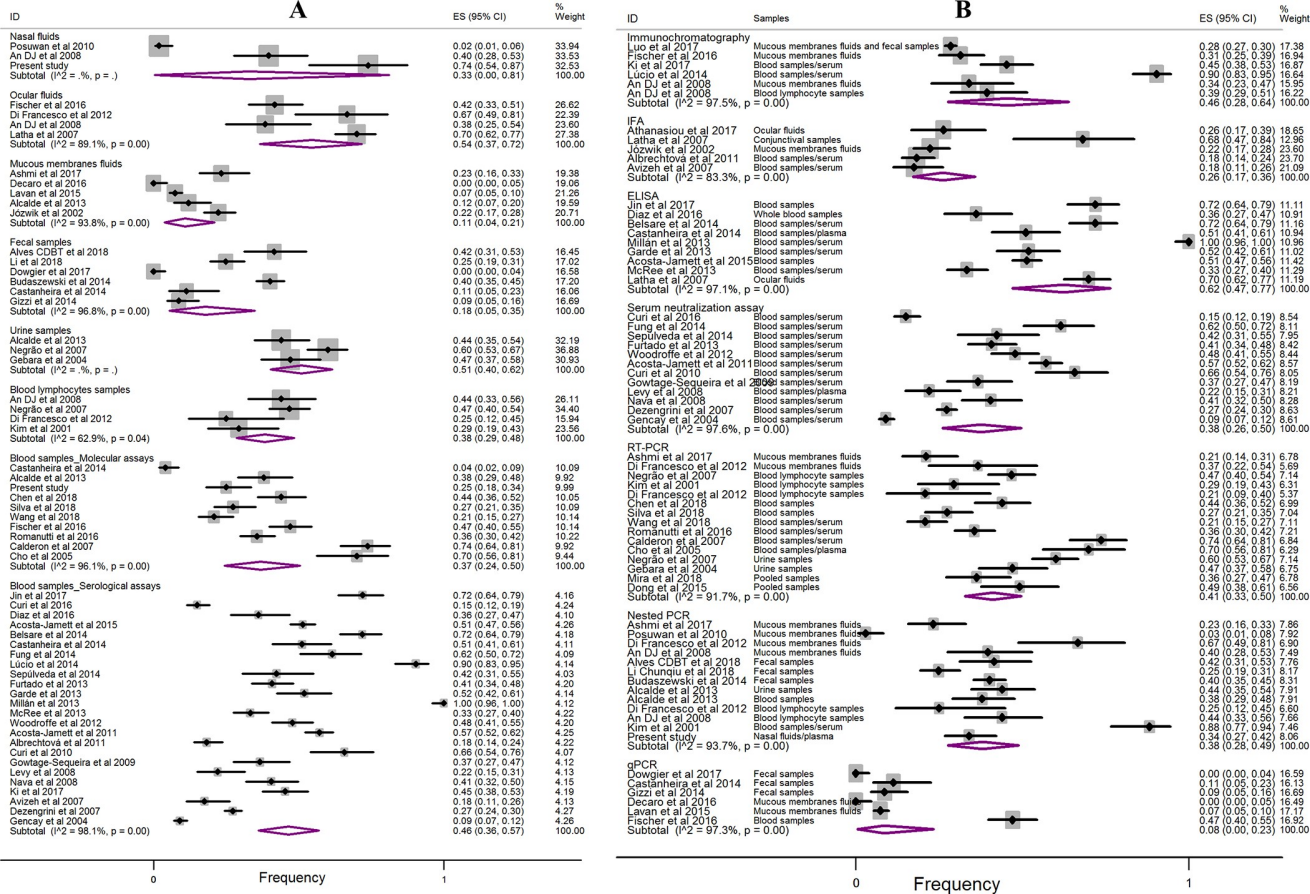
Fig 4A shows the forest plot of CDV positivity according to the type of biological sample surveyed. For Fig 4B, the forest plot is related to the diagnostic method.

Another study question was related to the determination of the levels of CDV infection according to the type of biological sample analyzed in the laboratory. As a result, greater positivity was observed for samples from ocular fluids (54%, 95% CI: 37–72), urine (51%, 95% CI: 40–62) and blood (46%, 95% CI: 36–57 (serological assays)). For the other types of biological samples, reduced positive frequency rates were found in blood lymphocytes (38%, 95% CI: 29–48), blood (37%, 95% CI: 24–50 (molecular assays)) and nasal fluids (33%, 95% CI: 0–81) (Fig 4A). A lower frequency of viral infection was observed in fecal samples (18%, 95% CI: 5–35) and mucous fluid (11%, 95% CI: 4–21), which refers to the mixing of biological samples composed of nasal, ocular, oropharyngeal, oronasal and genital tract swabs.

In the forest plot (Fig 4B), the proportion of CDV positivity is shown, and the data are related to the diagnostic method used in conjunction with the type of biological sample. Of note, a higher proportion of positivity occurred when using the ELISA assays (62%, 95% CI: 47–77), IC (46%, 95% CI: 28–64), RT-PCR (41%, 95% CI: 33–50), nested PCR (38%, 95% CI: 28–49) and SN (38%, 95% CI: 26–50). However, a lower proportion of positive CDVs occurred when using quantitative PCR assays (8%, 95% CI: 0–23) and IFA (26%, 95% CI: 17–36).

**Fig 5:**
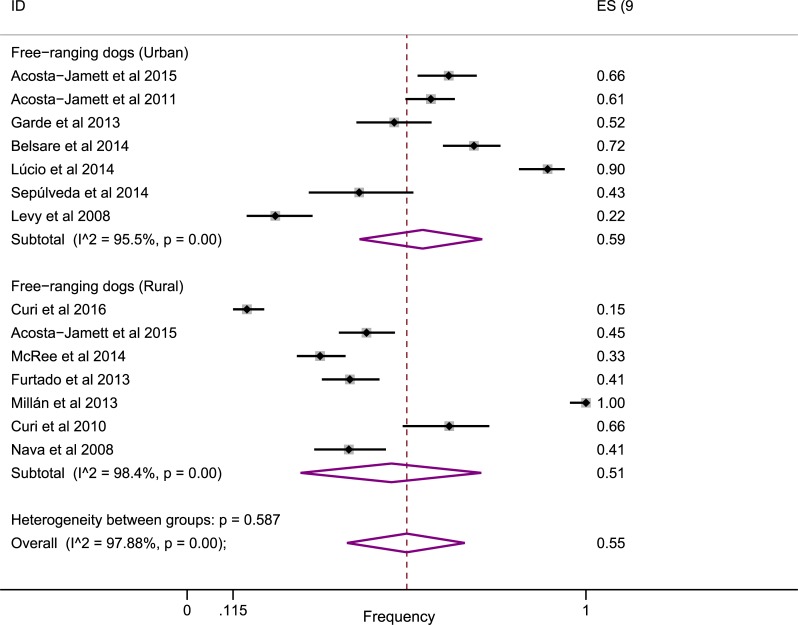
Forest plot showing the frequency of CDV positivity in relation to free-ranging dogs.

When the main behavioral factor was analyzed, a high proportion of positivity was observed in the free-ranging dogs (Fig 5). Thus, the proportion of CDV positivity was higher for free-ranging dogs (55%, 95% CI: 40–70) compared to the overall estimates of 37% (antigen surveys) and 46% (antibody surveys), which represented the pooled data of non-free-ranging and free-ranging animals.

For the analysis of the frequency of viral coinfection, only four studies [14,27,30,67] provided the necessary data for the calculation of the proportions. The common viral pathogens involved in CDV coinfection were canine parvovirus (35%, 95% CI: 21–49), canine adenovirus (4%, 95% CI: 1–10) and canine coronavirus (24%, 95% CI 15–34) (S1 Fig).

In the analysis of the positivity rate of CDV over time, the included studies ranged from 1998 to 2018. Thus, we analyzed whether there was any trend between the positivity rates and the year of collection of the biological samples. As a consequence, the results more closely approximated a visual steady trend, as shown in S2 Fig.

### A meta-analysis to evaluate risk factors associated with CDV positivity

**Fig 6:**
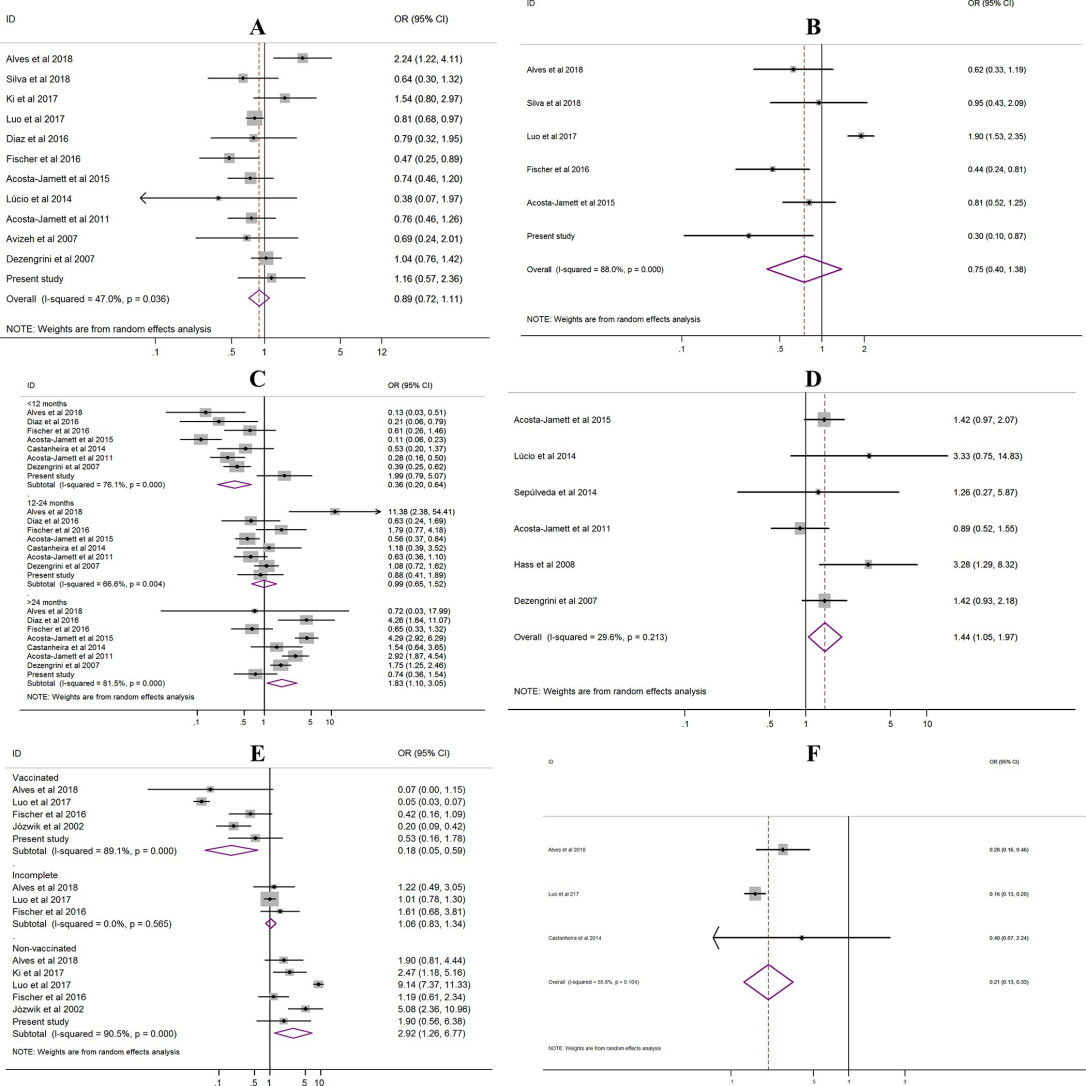
Forest plot of the odds ratio (OR) for CDV positivity in domestic dogs. A = gender, male versus female; B = purebred versus mixed-breed; C = age, <12 months, 12–24 months, and >24 months; D = free-ranging dogs versus non-free-ranging dogs; E = vaccinated versus incomplete and nonvaccinated; F = coinfection, CDV versus canine parvovirus.

For the purpose of testing potential risk factors associated with CDV positivity, the following variables were analyzed: gender; breed; age; free-ranging; vaccine status; and coinfection. In Fig 6, the results of the ORs for these variables are shown. In summary, a positive association with CDV positivity was observed in relation to the following variables: free-ranging dogs (OR = 1.44, 95% CI: 1.05–1.97); age of dogs >24 months (OR = 1.83, 95% CI: 1.10–3.05); and unvaccinated dogs (OR = 2.92, 95% CI: 1.26–6.77). In contrast, there was a negative association with vaccinated dogs (OR = 0.18, 95% CI: 0.05–0.59), dogs <12 months old (OR = 0.36, 95% CI: 0.20–0.64) and dogs that were coinfected with canine parvovirus (OR = 0.21, 95% CI: 0.13–0.33). The other variables, such as gender, breed and incomplete vaccination status, had no association.

### Sensitivity analysis and publication bias

When performing the sensitivity analysis to assess the weight of each individual study on the combined frequency through the removal of individual studies, there was no study that significantly affected the combined frequency (S4 Table). In addition, subgroup analyses were performed. For the majority of the results, high heterogeneity was observed (I^2^>75%). Low heterogeneity was found only in the subgroup of clinical signs (vomit, diarrhea and dermatological signs; I^2^ = 0.0). It should be emphasized that these results were expected, given that in observational epidemiological studies there is a considerable occurrence of diversity due to the study design, detection methodology and epidemiological variations.

In the analysis of publication bias, asymmetry of the funnel plot was noted for the CDV positivity frequency subgroups molecular surveys, serological surveys and free-ranging dogs (S3 Fig). However, when analyzing the asymmetry by Egger’s test, significant bias was observed only for the subgroup serological surveys (P = 0.02).

## Discussion

In this cross-sectional study and meta-analysis, frequencies and analysis of risk factors for CDV infection in domestic dogs were investigated. Interestingly, the results showed a high frequency of viral positivity obtained from serological and molecular assays. Therefore, we found that almost a third of suspected CD-infected and almost half of apparently healthy dogs were CDV-positive (33–46%; 95% CI: 23–57) (Fig 3A). These general data show the high likelihood of dogs being exposed to CDV throughout their lives and show their prominent role in the viral transmission chain [80]. In view of this, the importance of epidemiological studies of CDV is highlighted as it is a valuable tool in monitoring viral dissemination and in the development of animal public health strategies.

Among the viruses that affect dogs, CD is the most relevant disease after rabies due to its considerable dissemination and severity potential [81]. However, it has been shown that half of the CDV infections are subclinical or so mild that they do not require veterinary care [82]. However, mild disease may develop into severe disease in dogs, and in this case, the initial clinical condition, which is often restricted to fever, respiratory, ocular signs, apathy and inappetence, may result in severe impairment of the gastrointestinal tract (vomiting and diarrhea) and central nervous system (paraparesis or tetraparesis with sensitive ataxia and myoclonus) [5,80,81,83]. Thus, neurological signs may be progressive, and the onset of sequelae tends to generate an expectation of poor prognosis, which reflects a reduced survival rate. In this context, few studies have described the outcome of CDV-positive dogs [36,42, 71]. Here, we observed a fatality rate of 55% (95% CI: 47–64, I^2^: 0%) for animals with predominantly neurological signs, demonstrating how dangerous CD is.

When obtaining the frequencies of CDV infection in relation to clinical signs and types of biological samples, the factors associated with greater positivity were dogs with systemic, systemic-neurological and ocular signs in conjunction with samples of ocular fluids, blood and urine. The determination of which sample to analyze depends on the method of detection and the opportunity to collect the biological material representative of the evident clinical signs [84]. Thus, in a suspected case of CD, those animals with exuberant ocular and nasal secretions tend to provide good clinical material for screening since swabs of ocular and nasal secretions specimens are easy to obtain at an early stage of CDV infection [85]. For animals with early systemic signs, including fever, prostration and inappetence, the indicated specimen choice would be blood and/or urine. Some studies have verified that urine is a good biological sample for the detection of CDV RNA [36–38], and although the authors used RT-PCR, which is less sensitive than nested PCR, results continued to show its excellent application for laboratory diagnostic purposes.

As briefly mentioned, regional epidemiological variations of CDV may be based on study design, detection methodologies and epidemiological aspects, which include differences in the populations studied. All of these factors contribute to the variations in CDV frequency; there have been individual studies with positivity rates varying from 0 to 100% in regions of Italy [55,61] and Uganda [69], respectively. However, most of the articles reported a frequency of positivity between 30 and 50% (Fig 3A). In our experimental study, the lowest observed frequency (25%) in blood samples compared to the overall estimate (37%) may have occurred due to differences in the studied populations and/or due to the period of infection (Fig 4). Several dogs had already presented neurological impairment with clinical signs present several days prior; therefore, the possibility of finding viral RNA in the plasma was reduced, even when using nested PCR.

In the detection methodology, the serological tests included ELISA, IC, IFA and SN. The molecular assays included PCR and its variants. Currently, all of these test methodologies are financially accessible for use in the laboratory, but the IFA and PCR variants require reagents and equipment of higher financial cost, and because of this, their satisfactory use in the laboratory will depend on the number of samples to be examined. In addition, regarding the use of these assays in the present meta-analysis, higher positivity rates were reported from ELISA, IC and RT-PCR assays; therefore, in the future, data regarding laboratory accuracy should be investigated. For ELISA assays, only a few authors analyzed the sensitivity and specificity parameters, with indices varying from 93 to 100% and from 83 to 100% for sensitivity and specificity, respectively [53,86]. However, further studies are needed to better understand the diagnostic accuracy, including a higher number and varied types of biological samples. In the SN assay, known as the gold standard in antibody detection [87], the positivity rate of 38% (95% CI: 26–50) was lower to the global average, and approximately 1/2 of the seroepidemiological studies used SN. Regarding nested PCR, referred to as the gold standard in the diagnosis of CDV RNA, there was excellent sensitivity; however, the laboratory accuracy is susceptible to variation, including the sample collection during the clinical manifestations of acute CDV infection, the type of sample collected, the RNA extraction protocol and the primers used [88]. In these examples, it is not possible to estimate the probability of false-negative and/or false-positive results in our individual frequency estimates, but due to the robust global n-sample, this diagnostic bias tends to be reduced for the combined frequency estimate.

As a complementary investigation, we performed an analysis of the risk factors for CDV positivity. Consistent with the previous literature, we found no association between viral infection and the breed and gender of dogs, showing that regardless of whether dogs are male, female, purebred or mixed-breed, susceptibility to the etiological agent does not differ significantly between them [41,49,67,76,89]. In contrast, partial inconsistency with the literature was found in the negative association with pups [49,56,76]. Consequently, a higher risk of pups being affected by CD has been reported, but our results showed no association (OR = 0.59, 95% CI: 0.15–2.33). However, in the subgroup of pups without CD, there was a negative association between positivity (OR = 0.27, 95% CI: 0.16–0.46), indicating the vulnerability to CDV infection in this subgroup. In relation to adult dogs (> 24 months), the positive association observed was probably related to the fact that with the passage of time, the probability of exposure to the viral agent in the environment increases. In addition, another factor that increases the risk of infection is related to the behavioral factors of free-ranging or stray dogs. The free-ranging lifestyle of dogs likely means that they are not vaccinated and are constantly exposed to canine populations, which may justify the higher positivity of CDV in free-ranging dogs (55%) (Fig 5).

In the analyses of risk factors, it is important to highlight that it was impossible to perform ORs adjusted, such as grouping the vaccination status of the animals in relation to different ages and associating them with CDV infection by the fact of most of the selected studies were based on the inclusion of unvaccinated animals. Additionally, the absence of vaccination status in each region evaluated may have contributed to the existence of biases in the results obtained. Also, it is important to highlight the potential existence of vaccination status biases in the dog lifestyle (free-ranging dogs) results considering that there were no data in the literature in order to adjust the OR. Thus, it is interesting to mention that the OR would be influenced by the fact that free-ranging dogs, especially non-vaccinated dogs, tend to be more exposed to CDV because of the greater possibility of contact with other non-vaccinated dogs and eventually CDV infected, especially in urban environments. However, for two studies [32,53] among those included in the analyses of risk factors, we can infer the absence of biases of the vaccine status factor in the variables of age, race, sex and behavioral style of the animals in the regions of the Santa Cruz (Galapagos) and Cape Verde, considering that they are regions with no vaccination programs.

Domestic dogs are a source of CDV transmission for wildlife. This is because dogs acting as major reservoirs can infect and cause disease in wildlife [90,91]. This issue highlights the impact of dog diseases on wildlife conservation, as they enter these habitats and have contributed to the emergence of fatal CD outbreaks. Examples include CD epidemics in dogs and other wild species that have threatened populations of African lions (*Panthera leo*) in the Serengeti ecosystem and Ethiopian wolves (*Canis simensis*) [92,93]. Additionally, the diversity of animals susceptible to CDV infection is broad as shown in a systematic review by Martinez-Gutierrez [94] who did not include domestic dogs in their analysis. Interestingly, in that analysis, the taxonomic families with the largest number of existing studies were *Canidae* followed by *Felidae* and *Mustelidae*. The median seropositivity of CDV was 35.6%, 34.1% and 41.1% for *Canidae*, *Felidae* and *Mustelidae*, respectively [94]. These data, as well as our data, show how common the circulation of CDV is in these groups of animals.

This systematic review and meta-analysis had several strengths. First, due to recent observational studies, there was a need for an updated systematic review. Second, this seemed to be the first meta-analysis on the intended subject. Third, it was possible to conduct multiple analyses of relevant factors by subgroups for the present theme. Fourth, to eliminate antibody positivity from immunization in serological surveys, care was taken to collect data restricted to animals not vaccinated against CDV. Despite the strengths of our study, which generated enough power to implement a comprehensive analysis, there is still room for future improvements that will depend on the quality of the data from future, improved studies. There were some limitations in this meta-analysis that must be considered when interpreting the results. First, heterogeneity was observed in most of the analyses; therefore, heterogeneity in the subgroup was still relatively significant, and the results should be interpreted with caution. Second, there was a partial possibility of publication bias due to the asymmetrical funnel plot and the result of Egger’s test; in other words, there was the possibility that unpublished articles were not included in our metadata. Third, the results of our analysis were mainly based on unadjusted estimates, which may have led to some bias in the results. Thus a more accurate analysis would be possible if individual crude data were available.

## Conclusion

In summary, in the current meta-analysis (including our present study), the frequency rate of CDV positivity among molecular surveys was 33% (95% CI: 23–43) and among serological surveys, the rate was 46% (95% CI: 36–57), with considerable regional epidemiological variations in clinical signal parameters, biological samples, detection methods and animal lifestyle. Variables of adult (>24 months), free-ranging and unvaccinated dogs were found to be predictors of CDV infection. In contrast, complete vaccination, coinfection with parvovirus and pups (<12 months) had a negative association. Therefore, considering the high frequency of CDV positivity found across almost all variables analyzed, it is necessary to plan immediate and continuous mitigation strategies aiming to reduce infection levels, especially in certain endemic localities. In view of this, constant epidemiological surveillance, control of street dog populations, and more knowledge and access for dog owners to the complete CDV vaccine scheme is essential.

## Supporting information

S1 File PRISMA 2009 checklist. (DOC)

S2 File Critical appraisal checklist for the quality assessment of studies. (DOCX)

S3 File Crude data extracted for the generation of the meta-analysis. (XLSX)

S1 Table List of excluded full text papers with proper justification. (DOCX)

S2 Table The characteristics of the studies included in the meta-analysis. (DOCX)

S3 Table Frequency of CDV infection regarding the regions of origin of the articles. (DOCX)

S4 Table The sensitivity analysis to estimate of the frequency of CDV positivity. (DOCX)

S1 Fig Frequency of coinfection between CDV with canine parvovirus (CPV), canine adenovirus (CAdV) and canine coronavirus (CCoV). (TIF)

S2 Fig CDV positivity by year of biological sample collection. The variation corresponds to the 95% CI.
(TIF)

S3 Fig Funnel plot for CDV frequencies in subgroups molecular surveys (A), serological surveys (B) and free-ranging dogs (C).
(TIF)
