# Supplementary material for: Molecular and serological surveys of canine distemper virus: A meta-analysis of cross-sectional studies
Source: PLoS One. 2019 May 29;14(5):e0217594. doi: 10.1371/journal.pone.0217594 (PMC6541297; doi:10.1371/journal.pone.0217594)
Supplement: S2 File — (DOCX) [file pone.0217594.s002.docx]

**S2 File. Critical appraisal checklist for Quality Assessment of Studies**

| S. no | Studies to address | Criteria items and the adopted score |
| --- | --- | --- |
| MAJOR CRITERIA | | |
| 1 | Inclusion of study participants- Whether case definition of canine distemper is mentioned | A. Case definition for samples from dogs clinically suspected of distemper = 1  B. No case definition/ Unclear = 0 |
| 2 | Whether any sampling method for inclusion of patients is adopted in the study? | A. Included all/ Systematically/ Randomly = 1 B. Unknown/ Unclear = 0 |
| 3 | Measurement of disease condition | A. antigen detection/viral isolation/PCR = 1  B. Serological = 0.5 (‘A’ for sero-survey)  C. No lab test/Unclear = 0 |
| 4 | Whether vaccination status is mentioned in the paper? | A. Yes = 1  B. Unclear/No = 0 |
| MINOR CRITERIA | | |
| 1 | Whether age distribution of cases is given? | A. Both in tested and positive = 1  B. Only in tested or positive = 0.5  C. Not given/Unclear = 0 |
| 2 | Whether study is not limited any speciality setting? | A. Yes = 1  B. No/ Unclear = 0 |
| 3 | Whether CFR is reported? | A. Yes = 1  B. No/ Unclear = 0 |

**Overall risk of bias assessment**

| **QUALITY** | **RISK OF BIAS** | **CRITERIA** |
| --- | --- | --- |
| HIGH | LOW | ALL MAJOR = 1 + ALL MINOR = 0 OR ONLY ONE MINOR = 0 |
| MODERATE | MODERATE | 2 MAJOR = 1 + LESS THAN ONE MINOR = 0 |
| LOW | HIGH | 2 MAJOR = 0 + TWO MINOR = 0 |
| VERY LOW | VERY HIGH | NO MAJOR = 1 + NO MINOR = 1 |

| **ID** | **Major Criteria** | | | | **Minor Criteria** | | | **Quality** |
| --- | --- | --- | --- | --- | --- | --- | --- | --- |
|  | **1** | **2** | **3** | **4** | **1** | **2** | **3** |  |
| An DJ et al 2008 | A | A | A/B | B | B | A | B | Moderate |
| Alves CDBT et al 2018 | A | A | A | A | A | B | B | High |
| Ashmi JM et al 2017 | A | A | A | B | B | A | B | Moderate |
| Castanheira P et al 2014 | - | A | A | A | B | - | - | High |
| Gencay A et al 2004 | - | A | A | B | C | - | - | Moderate |
| McRee A et al 2014 | - | A | A | A | C | - | - | High |
| Li C et al 2018 | A | A | A | A | B | B | B | High |
| Gebara CMS et al 2004 | A | A | A | B | C | A | B | Moderate |
| Negrão FJ et al 2007 | A | A | A | B | C | A | B | Moderate |
| Alcalde R et al 2013 | A | A | A | A | C | A | B | High |
| Romanutti C et al 2015 | B | A | A | A | B | A | B | Moderate |
| Calderon MG et al 2007 | B | A | A | B | B | A | B | Moderate |
| Jin Y et al 2017 | - | A | A | A | C | A | B | High |
| Fischer CDB et al 2016 | A | A | A | A | A | A | A | High |
| Curi NHA et al 2016 | - | A | A | A | C | A | - | High |
| Acosta-Jamett G et al 2015 | - | A | A | A | A | A | - | High |
| Fung HL et al 2014 | - | A | A | B | C | A | B | Moderate |
| Sepúlveda MA et al 2014 | - | A | A | A | C | A | - | High |
| Furtado MM et al 2013 | - | A | A | A | C | A | - | High |
| Woodroffe R et al 2012 | - | A | A | A | C | A | - | High |
| Acosta-Jamett G et al 2011 | - | A | A | A | A | A | - | High |
| Gowtage-Sequeira et al 2009 | A | A | A | A | A | A | B | High |
| Navas AFD et al 2008 | - | A | A | A | C | A | - | High |
| Avizeh R et al 2007 | - | A | A | A | A | A | - | High |
| Diaz NM et al 2016 | - | A | A | A | A | A | - | High |
| Budaszewski RF et al 2014 | A | A | A | A | A | A | B | High |
| Dowgier G et al 2017 | A | A | A | B | C | B | B | Moderate |
| Hass R et al 2008 | - | A | A | A | A | A | - | High |
| Levy JK et al 2008 | - | A | A | A | B | A | - | High |
| Józwik A et al 2002 | A | A | A | A | A | A | - | High |
| Albrechtová K et al 2011 | - | A | A | B | A | A | - | Moderate |
| Lavan R et al 2015 | - | A | A | B | C | B | - | Moderate |
| Decaro N et al 2016 | - | A | A | B | C | B | B | Moderate |
| Gizzi ABR et al 2014 | A | A | A | B | C | B | B | Moderate |
| Kim YH et al 2001 | A | A | A | B | C | A | B | Moderate |
| Dezengrini R et al 2007 | - | A | A | A | A | A | - | High |
| Garde E et al 2013 | A | A | A | A | C | A | - | High |
| Curi NHA et al 2010 | - | A | A | A | C | A | - | High |
| Luo H et al 2017 | B | A | A | A | A | A | B | Moderate |
| Belsare AV et al 2014 | - | A | A | A | B | A | - | High |
| Millán J et al 2013 | - | A | A | A | C | A | - | High |
| Athanasiou et al 2017 | A | A | A | A | A | A | B | High |
| Chen et al 2018 | A | A | A | B | C | A | B | Moderate |
| Mira et al 2018 | A | A | A | A | B | A | A | High |
| Di Francesco et al 2012 | B | A | A | A | C | B | B | Moderate |
| Dong et al 2015 | B | A | A | B | C | A | B | Low |
| Latha et al 2007 | A | A | A | A | A | A | B | High |
| Cho et al 2005 | A | A | A | B | C | A | B | Moderate |
| Lúcio et al 2014 | A | A | B | B | C | A | B | Low |
| Posuwan et al 2010 | A | A | A | B | C | A | B | Moderate |
| Wang et al 2018 | A | A | A | B | C | A | B | Moderate |
| Ki et al 2017 | B | A | A | A | A | A | B | Moderate |
| Silva et al 2018 | A | A | A | A | C | B | B | High |
