# Supplementary material for: Molecular and serological surveys of canine distemper virus: A meta-analysis of cross-sectional studies
Source: PLoS One. 2019 May 29;14(5):e0217594. doi: 10.1371/journal.pone.0217594 (PMC6541297; doi:10.1371/journal.pone.0217594)
Supplement: S1 Table — (DOCX) [file pone.0217594.s004.docx]

**S1Table**

Descriptors: ‘Canine distemper virus’ OR ‘domestic dogs’ OR ‘Viruses in dogs’ from 1998 to 2018

Research Results PubMed: 110; SciELO: 20; SCIENCEDIRECT: 255 (original research/news/short communications /others). [Duplicate= 32]. Adittional records *n*= 54. N total= 439

In green: selected paper (n=53)

In yellow: paper excluded in the last selection step (n=77)

**In Bold: duplicate studies (n=33)**

| **N°** | **TITLE** | **JUSTIFICATION EXCLUSION** | **PERIODIC** |
| --- | --- | --- | --- |
| **PUBMED** | | | |
| 01 | Phylogenetic analysis of the wild-type strains of canine distemper virus circulating in the United States | Samples known to be positive for CDV | Virol J |
| 02 | Phylogenetic analysis of canine distemper virus in South African wildlife | Samples mainly of non-domestic dogs | PLoS ONE |
| 03 | Prevalence and genome characteristics of canine astrovirus in southwest China | Cases of CDV coinfection were only investigated in samples of canine astrovirus positive | J Gen Virol |
| **04** | **Identification of enteric viruses circulating in a dog population with low vaccine coverage** |  | **Braz J Microbiol** |
| **05** | **A multiplex TaqMan real-time PCR for detection and differentiation of four antigenic types of canine parvovirus in China** | **Detection of canine parvovirus** | **Mol Cell Probes** |
| 06 | Canine distemper viral infection threatens the giant panda population in China |  | Oncotarget |
| 07 | Serologic response to canine distemper vaccination in captive Linnaeus's two-toed sloths (*choloepus didactylus*) after a fatal canine distemper virus outbreak | Non-domestic dog | J Zoo Wildl Med |
| 08 | 6-methylmercaptopurine riboside, a thiopurine nucleoside with antiviral activity against canine distemper virus in vitro. | Antiviral analysis. In vitro study | Virol J |
| 09 | Viral gut metagenomics of sympatric wild and domestic canids, and monitoring of viruses: Insights from an endangered wolf population | Pool of samples from domestic dogs | Eco Evol |
| 10 | Pathological findings in the red fox (Vulpes vulpes), stone marten (Martes foina) and raccoon dog (Nyctereutes procyonoides), with special emphasis on infectious and zoonotic agents in Northern Germany | Non-domestic dog | PLoS ONE |
| 11 | Multiple-host pathogens in domestic hunting dogs in Nicaragua’s Bosawás Biosphere Reserve | Without definition of vaccine status | Acta Trop |
| 12 | Advances in canine distemper virus pathogenesis research: a wildlife perspective | Non-domestic dog | J Gen Virol |
| **13** | **Lethal distemper in badgers (Meles meles) following epidemic in dogs and wolves** | **Non-domestic dog** | **Infect Genet Evol** |
| **14** | **Enhanced immunosurveillance for animal morbilliviruses using vesicular stomatitis virus (VSV) pseudotypes.** | **Non-domestic dog (domestic livestock and wild ruminants)** | **Vaccine** |
| 15 | Isolation and phylogenetic analysis of canine distemper virus among domestic dogs in Vietnam | Unspecified sample size | J Vet Med Sci |
| 16 | Diversified Anchoring Features the Peptide Presentation of DLA-88*50801: First Structural Insight into Domestic Dog MHC Class I | Non-epidemiological study | J Immunol |
| 17 | Exposure of Free-Ranging Wild Carnivores and Domestic Dogs to Canine Distemper Virus and Parvovirus in the Cerrado of Central Brazil. | Reduced sample size | Ecohealth |
| 18 | Characterization of a novel Canine distemper virus causing disease in wildlife | Non-domestic dog (raccoons and gray foxes) | J Vet Diagn Invest |
| 19 | Controversial results of therapy with mesenchymal stem cells in the acute phase of canine distemper disease | Therapy and treatment | Genet Mol Res |
| 20 | Diversity of susceptible hosts in canine distemper virus infection: a systematic review and data synthesis | Review on non-dog hosts | BMC Vet Res |
| **21** | **Phylogenetic analysis of canine distemper virus in South America clade 1 reveals unique molecular signatures of the local epidemic** |  | **Infect Genet Evol** |
| **22** | **Circovirus in domestic and wild carnivores: An important opportunistic agent?** | **Do not discriminate between suspected CDV animals** | **Virology** |
| 23 | Prevalence and risk factors for viral exposure in rural dogs around protected areas of the Atlantic forest |  | BMC Vet Res |
| 24 | Sequencing of emerging canine distemper virus strain reveals new distinct genetic lineage in the United States associated with disease in wildlife and domestic canine populations | Pool of samples from domestic dogs and fox | Virol J |
| **25** | **RT-PCR and sequence analysis of the full-length fusion protein of Canine Distemper Virus from domestic dogs** |  | **J Virol Methods** |
| 26 | Patterns of Exposure of Iberian Wolves (Canis lupus) to Canine Viruses in Human-Dominated Landscapes | Non-domestic dogs (Iberian Wolves) | Ecohealth |
| 27 | Cross-species transmission of canine distemper virus—an update | Review | One Health |
| 28 | Clinical and molecular investigation of a canine distemper outbreak and vector-borne infections in a group of rescue dogs imported from Hungary to Switzerland | Case report; reduced sample size | BMC Vet Res |
| **29** | **Molecular phylogeography of canine distemper virus: Geographic origin and global spreading** | **Database for molecular phylogeography of CDV** | **Mol Phylogenet Evol** |
| **30** | **Epidemiology of canine distemper and canine parvovirus in domestic dogs in urban and rural areas of the Araucanía region in Chile** |  | **Vet Microbiol** |
| 31 | Parasitology and serology of free-ranging coyotes (canis latrans) in north carolina, USA | Non-domestic dogs | J Wildl Dis |
| 32 | Demographic characteristics and infectious diseases of a population of American black bears in Humboldt County, California | Non-domestic dogs (American black bears [Ursus americanus]) | Vector Borne Zoonotic Dis |
| 33 | Serological detection of infection with canine distemper virus, canine parvovirus and canine adenovirus in communal dogs from Zimbabwe |  | J S Afr Vet Assoc |
| 34 | Dynamics of a morbillivirus at the domestic-wildlife interface: Canine distemper virus in domestic dogs and lions | Domestic dog data included CDV serology data - A Bayesian state–space model for transmission of CDV between dog and lion | Proc Natl Acad Sci USA |
| 35 | Phylogenetic analysis of canine distemper virus in domestic dogs in Nanjing, China | Only phylogenetic analysis of CDV | Arch Virol |
| 36 | Estimating the potential impact of canine distemper virus on the Amur tiger population (Panthera tigris altaica) in Russia | Model was used to simulate the impact of CDV in non-domestic dog (Amur tigers (Panthera tigris altaica) | PLoS ONE |
| **37** | **Canine distemper outbreak in raccoons suggests pathogen interspecies transmission amongst alien and native carnivores in urban areas from Germany** | **Non-domestic dog (raccoons)** | **Vet Microbiol** |
| 38 | Epidemiology of viral pathogens of free-ranging dogs and Indian foxes in a human-dominated landscape in central India |  | Transbound Emerg Dis |
| 39 | Parasitology, virology, and serology of free-ranging coyotes (Canis latrans) from central Georgia, USA | Non-domestic dog (coyotes [Canis latrans]) | J Wildl Dis |
| **40** | **Phylogenetic evidence of a new canine distemper virus lineage among domestic dogs in Colombia, South America** |  | **Vet Microbiol** |
| 41 | Molecular and serological surveillance of canine enteric viruses in stray dogs from Vila do Maio, Cape Verde |  | BMC Vet Res |
| **42** | **Domestic dog health worsens with socio-economic deprivation of their home communities** |  | **Acta Trop** |
| 43 | Invasive American mink: linking pathogen risk between domestic and endangered carnivores |  | Ecohealth |
| 44 | Using the ferret model to study morbillivirus entry, spread, transmission and cross-species infection | Non-domestic dog (ferret model) | Curr Opin Virol |
| 45 | Arctic lineage-canine distemper virus as a cause of death in Apennine wolves (Canis lupus) in Italy | Non-domestic dog (Apennine wolves) | PLoS ONE |
| 46 | Canine distemper virus (CDV) in another big cat: should CDV be renamed carnivore distemper virus? | Commentary | MBio |
| 47 | Canine distemper virus: an emerging disease in wild endangered Amur tigers (Panthera tigris altaica) | Non-domestic dog (Amur tigers [Panthera tigris altaica]) | MBio |
| **48** | **High prevalence of antibodies against canine adenovirus (CAV) type 2 in domestic dog populations in South Africa precludes the use of CAV-based recombinant rabies vaccines** | **Non-CDV study** | **Vaccine** |
| 49 | Rabies, canine distemper, and canine parvovirus exposure in large carnivore communities from two Zambian ecosystems | Reduced sample size | Vector Borne Zoonotic Dis |
| 50 | Serosurvey for selected viral infections in free-ranging jaguars (Panthera onca) and domestic carnivores in Brazilian Cerrado, Pantanal, and Amazon |  | J Wildl Dis |
| 51 | Serosurvey of dogs for human, livestock, and wildlife pathogens, Uganda |  | Emerg Infect Dis |
| 52 | The fusion protein signal-peptide-coding region of canine distemper virus: a useful tool for phylogenetic reconstruction and lineage identification | Study for phylogenetic analysis | PLoS ONE |
| 53 | Deduced sequences of the membrane fusion and attachment proteins of canine distemper viruses isolated from dogs and wild animals in Korea | Study for phylogenetic analysis | Virus Genes |
| 54 | Phylogenetic analysis of the haemagglutinin gene of canine distemper virus strains detected from giant panda and raccoon dogs in China | Non-domestic dog (giant panda and raccoon dogs) | Virol J |
| 55 | Effects of body weight on antibody titers against canine parvovirus type 2, canine distemper virus, and canine adenovirus type 1 in vaccinated domestic adult dogs | Dog vaccinated | Can J Vet Res |
| 56 | Rabies virus and canine distemper virus in wild and domestic carnivores in Northern Kenya: are domestic dogs the reservoir? | Pooled data about sero-prevalence | EcoHealth |
| 57 | Antagonistic pleiotropy and fitness trade-offs reveal specialist and generalist traits in strains of canine distemper virus | Non-epidemiological study | PLoS ONE |
| 58 | Pathogens of wild maned wolves (Chrysocyon brachyurus) in Brazil | Non-domestic dog (Chrysocyon brachyurus) | J Wildl Dis |
| 59 | Exposure to selected Pathogens in to selected pathogens in Geoffroy's cats and domestic carnivores from central Argentina | Study with a sample size <50 | J Wildl Dis |
| **60** | **Occurrence and geographical distribution of Canine Distemper Virus infection in red foxes (Vulpes vulpes) of Saxony-Anhalt, Germany** | **Non-domestic dog (Red foxes [Vulpes vulpes])** | **Vet Microbiol** |
| 61 | Emergence of canine distemper virus strains with modified molecular signature and enhanced neuronal tropism leading to high mortality in wild carnivores. | Study for phylogenetic analysis; Only one sample from domestic dog | Vet Pathol |
| 62 | Serosurvey of free-ranging Amur tigers in the Russian Far East | Study with a sample size <50 | J Wildl Dis |
| 63 | Contact with domestic dogs increases pathogen exposure in endangered African wild dogs (Lycaon pictus) |  | PLoS ONE |
| **64** | **Development of a combined canine distemper virus specific RT-PCR protocol for the differentiation of infected and vaccinated animals (DIVA) and genetic characterization of the hemagglutinin gene of seven Chinese strains demonstrated in dogs** | **Development of RT-PCR protocol with positive samples for CDV** | **J Virol Methods** |
| 65 | Pathogenesis and phylogenetic analyses of canine distemper virus strain ZJ7 isolate from domestic dogs in China | Study for the isolation of CDV with phylogenetic analysis | Virol J |
| 66 | Canine distemper spillover in domestic dogs from urban wildlife | Review | Vet Clin North Am Small Anim Pract |
| **67** | **Susceptibility of carnivore hosts to strains of canine distemper virus from distinct genetic lineages** | **Phylogenetic analysis with strain CDV positive** | **Vet Microbiol** |
| **68** | **Inhibition of viral RNA synthesis in canine distemper virus infection by proanthocyanidin A2** | **In vitro antiviral activity against CDV** | **Antiviral Res** |
| **69** | **Occurrence of filaria in domestic dogs of Samburu pastoralists in Northern Kenya and its associations with canine distemper** |  | **Vet Parasitol** |
| 70 | Domestic dog origin of canine distemper virus in free-ranging wolves in Portugal as revealed by hemagglutinin gene characterization | Phylogenetic analysis | J Wildl Dis |
| **71** | **Urban domestic dog populations as a source of canine distemper virus for wild carnivores in the Coquimbo region of Chile** |  | **Vet Microbiol** |
| 72 | Phylogenetic analysis of Austrian canine distemper virus strains from clinical samples from dogs and wild carnivores | Phylogenetic analysis | Vet Rec |
| 73 | Exposure to infectious agents in dogs in remote coastal British Columbia: Possible sentinels of diseases in wildlife and humans | Reduced sample size | Can J Vet Res |
| 74 | Comparison of canine distemper viruses in domestic dogs and wild raccoon dogs in South Korea | Phylogenetic analysis on diagnosed animals with CDV infection | Vet Rec |
| 75 | Multiplex Amplification Refractory Mutation System Polymerase Chain Reaction (ARMS-PCR) for diagnosis of natural infection with canine distemper virus | No distinction of vaccination history | Virol J |
| **76** | **Demography of domestic dogs in rural and urban areas of the Coquimbo region of Chile and implications for disease transmission** | **Data previously used** | **Prev Vet Med** |
| 77 | Seroprevalences to viral pathogens in free-ranging and captive cheetahs (Acinonyx jubatus) on Namibian Farmland | Non-domestic dog (captive cheetahs [Acinonyx jubatus]) | Clin Vaccine Immunol |
| 78 | Epidemiology, pathology, and genetic analysis of a canine distemper epidemic in Namibia |  | J Wildl Dis |
| 79 | Virologic survey of dogs with naturally acquired idiopathic conjunctivitis | Unavailable data | J Am Vet Med Assoc |
| **80** | **Phylogenetic analysis of the haemagglutinin gene of canine distemper virus strains detected from breeding foxes, raccoon dogs and minks in China** | **Non-domestic dog** | **Vet Microbiol** |
| 81 | First evidence of canine distemper in Brazilian free-ranging felids |  | Ecohealth |
| 82 | Antibody titers against canine distemper virus in unvaccinated rural dogs from Ahvaz, Iran |  | Pak J Biol Sci |
| **83** | **Multi-host pathogens and carnivore management in southern Africa** | **Non-domestic dog** | **Comp Immunol Microbiol Infect Dis** |
| **84** | **Disease threats to the endangered Iberian lynx (Lynx pardinus)** | **Reduced sample size** | **Vet J** |
| 85 | Canine distemper virus in wild ferret-badgers of Taiwan | Non-domestic dog | J Wildl Dis |
| 86 | Serosurvey of pathogens in domestic dogs on the border of Noël Kempff Mercado National Park, Bolivia |  | J Zoo Wildl Med |
| 87 | Pathogen evolution and disease emergence in carnivores | Phylogenetic and molecular evolution analysis on CDV genes | Proc Biol Sci |
| **88** | **Detection by RT-PCR and genetic characterization of canine distemper virus from vaccinated and non-vaccinated dogs in Argentina** |  | **Vet Microbiol** |
| 89 | Exposure of free-ranging maned wolves (Chrysocyon brachyurus) to infectious and parasitic disease agents in the Noël Kempff Mercado National Park, Bolivia | Non-domestic dog (wolves [Chrysocyon brachyurus]) | J Zoo Wildl Med |
| 90 | Demography, hunting ecology, and pathogen exposure of domestic dogs in the Isoso of Bolivia | No description of crude CDV infection data | Conserv Biol |
| 91 | Canine vaccination-providing broader benefits for disease control | Review | Vet Microbiol |
| **92** | **Immunohistochemical detection of antigens of distemper, adenovirus and parainfluenza viruses in domestic dogs with pneumonia** | **Reduced sample size** | **J Comp Pathol** |
| **93** | **Phylogenetic and restriction fragment length polymorphism analyses of hemagglutinin (H) protein of canine distemper virus isolates from domestic dogs in Japan** | **Phylogenetic analysis** | **Virus Res** |
| 94 | Serosurvey of viral infections in free-ranging Namibian cheetahs (Acinonyx jubatus) | Non-domestic dog (cheetahs [Acinonyx jubatus]) | J Wildl Dis |
| 95 | Antibodies to canine and feline viruses in spotted hyenas (Crocuta crocuta) in the Masai Mara National Reserve | Non-domestic dog (spotted hyenas [Crocuta crocuta]) | J Wildl Dis |
| 96 | Clinical and serological response of wild dogs (Lycaon pictus) to vaccination against canine distemper, canine parvovirus infection and rabies | Non-domestic dog (wild dogs [Lycaon pictus]) | J S Afr Vet Assoc |
| 97 | Canine distemper in terrestrial carnivores: a review | Review | J Zoo Wildl Med |
| 98 | Antibodies to selected canine pathogens and infestation with intestinal helminths in golden jackals (Canis aureus) in Israel | Non-domestic dog (golden jackals [Canis aureus]) | Vet J |
| **99** | **Contact rates between wild and domestic canids: no evidence of parvovirus or canine distemper virus in crab-eating foxes** | **No vaccination status specified** | **Vet Microbiol** |
| 100 | Hemagglutinin genotype profiles of canine distemper virus from domestic dogs in Japan | Detection of lineages for the study of genotyping | Arch Virol |
| 101 | Serologic survey of selected viral agents in recently captured wild North American river otters (Lontra canadensis) | Non-domestic dog (Lontra [Lutra] canadensis) | J Zoo Wildl Med |
| 102 | Antibodies to CD9, a tetraspan transmembrane protein, inhibit canine distemper virus-induced cell-cell fusion but not virus-cell fusion | Non-epidemiological study | J Virol |
| **103** | **Epizootiological investigations of canine distemper virus in free-ranging carnivores from Germany** | **Non-domestic dog (red foxes, stone martens, raccoons)** | **Vet Microbiol** |
| **104** | **Serological and demographic evidence for domestic dogs as a source of canine distemper virus infection for Serengeti wildlife** | **Pooled data** | **Vet Microbiol** |
| **105** | **Morbillivirus infections, with special emphasis on morbilliviruses of carnivores** | **Review** | **Vet Microbiol** |
| 106 | Genotypes of canine distemper virus determined by analysis of the hemagglutinin genes of recent isolates from dogs in Japan | Phylogenetic analysis | J Clin Microbiol |
| 107 | Rapid and sensitive detection of immunoglobulin M (IgM) and IgG antibodies against canine distemper virus by a new recombinant nucleocapsid protein-based enzyme-linked immunosorbent assay | Study with a sample size <50 | J Clin Microbiol |
| **108** | **Genetic characterization of canine distemper virus in Serengeti carnivores** | **Phylogenetic analysis** | **Vet Immunol Immunopathol** |
| 109 | Survey on viral pathogens in wild red foxes (Vulpes vulpes) in Germany with emphasis on parvoviruses and analysis of a DNA sequence from a red fox parvovirus | Non-domestic dog (wild red foxes [Vulpes vulpes]) | Epidemiol Infect |
| 110 | A fast and simple one‑step duplex PCR assay for canine distemper virus (CDV) and canine coronavirus (CCoV) detection |  | Arch Virol |
| **SCIENCEDIRECT** | | | |
| 111 | Antigenic analysis of genetic variants of Canine distemper virus | Phylogenetic/vaccine analysis | Vet Microb |
| 112 | Epidemiology of canine distemper and canine parvovirus in domestic dogs in urban and rural areas of the Araucanía region in Chile |  | Vet Microb |
| 113 | RT-PCR and sequence analysis of the full-length fusion protein of Canine Distemper Virus from domestic dogs |  | J Virol Methods |
| 114 | Molecular characterization of complete genome of a canine distemper virus associated with fatal infection in dogs in Gabon, Central Africa | Laboratory confirmation of CDV of only a few samples | Virus Res |
| 115 | The role of toll-like receptor polymorphisms in susceptibility to canine distemper virus | Non-domestic dog | Mamm Biol |
| 116 | Canine distemper virus active infection in order Pilosa, family Myrmecophagidae, species Tamandua tetradactyla | Phylogenetic analysis in non-domestic dog | Vet Microbiol |
| 117 | Recombinant rabies virus expressing the H protein of canine distemper virus protects dogs from the lethal distemper challenge | Vaccination with chimeric virus against CDV | Vet Microbiol |
| 118 | Pathogenesis of canine distemper virus in experimentally infected raccoon dogs, foxes, and minks | Non-domestic dog (raccoon dogs, foxes, and minks) | Antiviral Res |
| 119 | Lethal distemper in badgers (Meles meles) following epidemic in dogs and wolves | Phylogenetic analysis | Infect, Genet Evol |
| 120 | Phylogenetic evidence of a new canine distemper virus lineage among domestic dogs in Colombia, South America |  | Vet Microbiol |
| 121 | Dog overpopulation and burden of exposure to canine distemper virus and other pathogens on Santa Cruz Island, Galapagos |  | Prev Vet Med |
| 122 | Recombinant canine distemper virus serves as bivalent live vaccine against rabies and canine distemper | Immunization studies in dogs | Vaccine |
| 123 | Phylogenetic analysis of canine distemper virus in South America clade 1 reveals unique molecular signatures of the local epidemic |  | Infect, Genet Evol |
| 124 | Occurrence of filaria in domestic dogs of Samburu pastoralists in Northern Kenya and its associations with canine distemper | Does not mention whey status of vaccination in dog | Vet Parasitol |
| 125 | New viruses associated with canine gastroenteritis | Review | Vet J |
| 126 | Epidemiology of canine distemper virus in wild raccoon dogs (Nyctereutes procyonoides) from South Korea | Non-domestic dog (Raccoon dogs[Nyctereutes procyonoides]) | Comp Immunol, Microbiol Infect Dis |
| 127 | A model-based approach for investigation and mitigation of disease spillover risks to wildlife: Dogs, foxes and canine distemper in central India | Biological background for model purpose | Ecol Model |
| 128 | Phylogenetic and restriction fragment length polymorphism analyses of hemagglutinin (H) protein of canine distemper virus isolates from domestic dogs in Japan | Phylogenetic analysis | Virus Res |
| 129 | Multiple-host pathogens in domestic hunting dogs in Nicaragua’s Bosawás Biosphere Reserve | Does not mention whey status of vaccination in dog | Acta Trop |
| 130 | Genotyping of canine distemper virus strains circulating in Brazil from 2008 to 2012 |  | Virus Res |
| 131 | The recombinant EHV-1 vector producing CDV hemagglutinin as potential vaccine against canine distemper | Vector producing CDV hemagglutinin for vaccine against CDV | Microb Pathog |
| 132 | Urban domestic dog populations as a source of canine distemper virus for wild carnivores in the Coquimbo region of Chile |  | Vet Microbiol |
| 133 | Molecular phylogeography of canine distemper virus: Geographic origin and global spreading | Database for molecular phylogeography of CDV | Mol Phylogenet Evol |
| 134 | Establishment of canine and feline cells expressing canine signaling lymphocyte activation molecule for canine distemper virus study | In vitro study | Vet Microbiol |
| 135 | Establishment of reverse transcription loop-mediated isothermal amplification for rapid detection and differentiation of canine distemper virus infected and vaccinated animals | Samples obtained from vaccinated dog farms | Infect, Genet Evol |
| 136 | Identification of enteric viruses circulating in a dog population with low vaccine coverage |  | Braz J Microbiol |
| 137 | Epizootic canine distemper virus infection among wild mammals | Non-domestic dog (Raccoon) | Vet Microbiol |
| 138 | Immunohistochemical Detection of Antigens of Distemper, Adenovirus and Parainfluenza Viruses in Domestic Dogs with Pneumonia |  | J Comp Pathol |
| 139 | Recombinant Newcastle disease viral vector expressing hemagglutinin or fusion of canine distemper virus is safe and immunogenic in minks | Attenuated CDV vaccines for mink, ferret and some wildlife species | Vaccine |
| 140 | Canine distemper outbreak in raccoons suggests pathogen interspecies transmission amongst alien and native carnivores in urban areas from Germany | Non-domestic dog (raccoons) | Vet Microbiol |
| 141 | Canine distemper virus neutralization activity is low in human serum and it is sensitive to an amino acid substitution in the hemagglutinin protein | Non-domestic dog | Virology |
| 142 | Emergence of canine distemper virus strains with two amino acid substitutions in the haemagglutinin protein, detected from vaccinated carnivores in North-Eastern China in 2012–2013 | Non-domestic dog (strains of CDV detected from vaccinated minks, foxes, and raccoon dogs) | Vet J |
| 143 | The administration of a single dose of a multivalent (DHPPiL4R) vaccine prevents clinical signs and mortality following virulent challenge with canine distemper virus, canine adenovirus or canine parvovirus | Vaccine challenge with CDV | Trials Vaccinol |
| 144 | Circulation of canine parvovirus among dogs living in human-wildlife interface in the Atlantic forest biome, Brazil | Detection of canine parvovirus | Heliyon |
| 145 | Inhibition of viral RNA synthesis in canine distemper virus infection by proanthocyanidin A2 | In vitro assay | Antiviral Res |
| 146 | Genotypic lineages and restriction fragment length polymorphism of canine distemper virus isolates in Thailand | Confirmed isolated CDV | Vet Microbiol |
| 147 | The identification of frequent variations in the fusion protein of canine distemper virus | Investigate the genetic status of circulating strains of CDV | Vet J |
| 148 | Circovirus in domestic and wild carnivores: An important opportunistic agent? | Do not discriminate between suspected CDV animals | Virology |
| 149 | Susceptibility of carnivore hosts to strains of canine distemper virus from distinct genetic lineages | Phylogenetic analysis with strain CDV positive | Vet Microbiol |
| 150 | Phylogenetic analysis of the haemagglutinin gene of canine distemper virus strains detected from breeding foxes, raccoon dogs and minks in China | Non-domestic dog (breeding foxes, raccoon dogs and minks in China) | Vet Microbiol |
| 151 | Evidence of two co-circulating genetic lineages of canine distemper virus in South America | Phylogenetic analysis with strain CDV positive | Virus Res |
| 152 | Heterogeneity within the hemagglutinin genes of canine distemper virus (CDV) strains detected in Italy | Phylogenetic analysis with strain CDV positive | Vet Microbiol |
| 153 | Dog nectin-4 is an epithelial cell receptor for canine distemper virus that facilitates virus entry and syncytia formation | Non-epidemiological study | Virology |
| 154 | Genetic diversity of Hungarian canine distemper virus strains | Specified only the number of samples analyzed | Vet Microbiol |
| 155 | Detection by RT-PCR and genetic characterization of canine distemper virus from vaccinated and non-vaccinated dogs in Argentina |  | Vet Microbiol |
| 156 | Serum-Neutralizing Antibody Responses to Canine Distemper Virus Vaccines in Domestic Ferrets (Mustela putorius furo) | Non-domestic dog (pet ferrets) | J Exotic Pet Med |
| 157 | In vitro inhibition of canine distemper virus by flavonoids and phenolic acids: Implications of structural differences for antiviral design | In vitro study | Res Vet Sci |
| 158 | Antiviral efficacy of EICAR against canine distemper virus (CDV) in vitro |  | Res Vet Sci |
| 159 | Serological and demographic evidence for domestic dogs as a source of canine distemper virus infection for Serengeti wildlife | Pooled data | Vet Microbiol |
| 160 | Development of a combined canine distemper virus specific RT-PCR protocol for the differentiation of infected and vaccinated animals (DIVA) and genetic characterization of the hemagglutinin gene of seven Chinese strains demonstrated in dogs | Development of RT-PCR protocol with positive samples for CDV | J Virol Methods |
| 161 | Early life DNA vaccination with the H gene of Canine distemper virus induces robust protection against distemper | Mink, vaccinated with DNA plasmids (haemagglutinin protein) of a vaccine strain CDV | Vaccine |
| 162 | Display of neutralizing epitopes of Canine parvovirus and a T-cell epitope of the fusion protein of Canine distemper virus on chimeric tymovirus-like particles and its use as a vaccine candidate both against Canine parvo and Canine distemper | VLP based vaccine | Vaccine |
| 163 | Fatal canine distemper infection in a pack of African wild dogs in the Serengeti ecosystem, Tanzania | Non-domestic dog (African wild dog [Lycaon pictus]) | Vet Microbiol |
| 164 | Variable transcription of pro- and anti-inflammatory cytokines in phocine lymphocytes following canine distemper virus infection | Study of phocine distemper virus in epidemics in seals | Vet Immunol Immunopathol |
| 165 | Immunization of Puppies in the Presence of Maternally Derived Antibodies Against Canine Distemper Virus | Non-epidemiologic study | J Comp Pathol |
| 166 | Humoral and cell-mediated immune responses in DNA immunized mink challenged with wild-type canine distemper virus | Immune response after DNA immunization with CDV | Vaccine |
| 167 | Molecular typing of a novel canine parvovirus type 2a mutant circulating in Italy | Canine parvovirus | Infect, Genetic Evol |
| 168 | Genetic characteristics of canine bocaviruses in Korean dogs | Canine bocavirus | Vet Microbiol |
| 169 | High prevalence of antibodies against canine adenovirus (CAV) type 2 in domestic dog populations in South Africa precludes the use of CAV-based recombinant rabies vaccines | Prevalence of antibodies against canine adenovirus in South African dogs. | Vaccine |
| 170 | A molecular survey for selected viral enteropathogens revealed a limited role of Canine circovirus in the development of canine acute gastroenteritis |  | Vet Microbiol |
| 171 | A multiplex TaqMan real-time PCR for detection and differentiation of four antigenic types of canine parvovirus in China | Samples positive for canine parvovirus | Mol Cell Probes |
| 172 | Whole genome sequence analysis of the arctic-lineage strain responsible for distemper in Italian wolves and dogs through a fast and robust next generation sequencing protocol | Phylogenetic analysis of CDV isolate of the Arctic-lineage strain | J Virol Methods |
| **173** | **Detection of respiratory viruses in shelter dogs maintained under varying environmental conditions** | **Does not mention whey status of vaccination against CDV** | **Braz J Microbiol** |
| 174 | Mamastrovirus 5 detected in a crab-eating fox (Cerdocyon thous): Expanding wildlife host range of astroviruses | Non-domestic dog (crab-eating fox [Cerdocyon thous]) | Compar Immunol, Microbiol Infect Dis |
| 175 | Epizootiological investigations of canine distemper virus in free-ranging carnivores from Germany | Non-domestic dog (red foxes, stone martens, raccoons) | Veter Microbiol |
| 176 | Occurrence and geographical distribution of Canine Distemper Virus infection in red foxes (Vulpes vulpes) of Saxony-Anhalt, Germany | Non-domestic dog (Red foxes [Vulpes vulpes]) | Vet Microbiol |
| 177 | Bait flavor preference and immunogenicity of ONRAB baits in domestic dogs on the Navajo Nation, Arizona | Non-epidemiological study of CDV | J Vet Beh: Clinical Applications and Research |
| 178 | Adaptation of canine distemper virus to canine footpad keratinocytes modifies polymerase activity and fusogenicity through amino acid substitutions in the P/V/C and H proteins | Non-epidemiological study of CDV | Virology |
| 179 | Phylogenetic analysis of the haemagglutinin gene of current wild-type canine distemper viruses from South Africa: Lineage Africa | Phylogenetic analysis of CDV | Vet Microbiol |
| 180 | Phylogenetic characterization of canine distemper virus isolates from naturally infected dogs and a marten in Korea | Phylogenetic analysis of CDV | Vet Microbiol |
| 181 | Retrospective biomolecular investigation of Coxiella burnetii and Leptospira spp. DNA in cases of abortion, stillbirth and neonatal mortality in dogs and cats | Non-epidemiological study of CDV | Top Comp Anim Med |
| 182 | Magnetic protein microbead-aided indirect fluoroimmunoassay for the determination of canine virus specific antibodies | Development of test for the detection of CDV | Biosens Bioelectro |
| 183 | Investigation of the presence of canine adenovirus (CAdV) in owned dogs in Northern Italy | Detection of CAdV in dog sample | Res Vet Sci |
| 184 | The effect of protected areas on pathogen exposure in endangered African wild dog (Lycaon pictus) populations | Non-domestic dog (African wild dogs [Lycaon pictus]) | Biol Cons |
| 185 | Molecular detection of Hepatozoon spp. in domestic dogs and wild mammals in southern Pantanal, Brazil with implications in the transmission route | Non-detection of CDV | Vet Parasitol |
| 186 | Host range and receptor utilization of canine distemper virus analyzed by recombinant viruses: Involvement of heparin-like molecule in CDV infection | Non-epidemiological study about sero-prevalence | Virology |
| 187 | Controversial results of the genetic analysis of a canine distemper vaccine strain | Non-epidemiological study about CDV sero-prevalence | Vet Microbiol |
| 188 | Domestic dog health worsens with socio-economic deprivation of their home communities |  | Acta Trop |
| 189 | A serological survey of avian origin canine H3N2 influenza virus in dogs in Korea | Non-CDV study | Vet Microbiol |
| 190 | Contact rates between wild and domestic canids: no evidence of parvovirus or canine distemper virus in crab-eating foxes | No vaccination status specified | Vet Microbiol |
| 191 | A minute virus of canines (MVC: canine bocavirus) isolated from an elderly dog with severe gastroenteritis, and phylogenetic analysis of MVC strains | Non-CDV study | Vet Microbiol |
| 192 | Membrane-bound SIV envelope trimers are immunogenic in ferrets after intranasal vaccination with a replication-competent canine distemper virus vector | Non-epidemiological study about CDV sero-prevalence | Virology |
| 193 | Prevention of rabies virus infection in dogs by a recombinant canine adenovirus type-2 encoding the rabies virus glycoprotein | Non-epidemiological study about CDV sero-prevalence | Microbes Infect |
| 194 | Prevalence of antibodies against canine distemper virus and canine parvovirus among foxes and wolves from Spain | Non-domestic dog | Vet Microbiol |
| 195 | Importance of canine distemper virus (CDV) infection in free-ranging Iberian lynxes (Lynx pardinus) | Non-domestic dog | Vet Microbiol |
| 196 | Infection with a Hepatozoon sp. closely related to Hepatozoon felis in a wild Pampas gray fox (Lycalopex – Pseudalopex – gymnocercus) co-infected with canine distemper virus | Non-domestic dog | Vet Parasitol |
| 197 | Canine vaccination—Providing broader benefits for disease control | Review | Vet Microbiol |
| 198 | Predation of wildlife by free-ranging domestic dogs in Polish hunting grounds and potential competition with the grey wolf | Non-epidemiological study about CDV sero-prevalence | Biol Cons |
| 199 | Demography of domestic dogs in rural and urban areas of the Coquimbo region of Chile and implications for disease transmission | Data previously used | Prev Vet Med |
| 200 | Expression of a foreign gene by recombinant canine distemper virus recovered from cloned DNAs | Non-epidemiological study about CDV sero-prevalence | Virus Res |
| 201 | Genetic characterization of canine distemper virus in Serengeti carnivores | Phylogenetic analyses | Vet Immunol Immunopathol |
| 202 | Development of a duplex real-time RT-qPCR assay to monitor genome replication, gene expression and gene insert stability during in vivo replication of a prototype live attenuated canine distemper virus vector encoding SIV gag | Non-epidemiological study about CDV sero-prevalence | J Virol Methods |
| 203 | Preference among 7 bait flavors delivered to domestic dogs in Arizona: Implications for oral rabies vaccination on the Navajo Nation | Non-epidemiological study about CDV sero-prevalence | J Vet Behav: Clinical Applications and Research |
| 204 | Early DNA vaccination of puppies against canine distemper in the presence of maternally derived immunity | CDV DNA vaccination of puppies | Vaccine |
| 205 | Human and domestic animal populations as a potential threat to wild carnivore conservation in a fragmented landscape from the Eastern Brazilian Amazon | Non-domestic dog (wild carnivore species) | Biol Cons |
| 206 | A new look at an old dog: Bonn-Oberkassel reconsidered | Non-epidemiological study about CDV sero-prevalence | J Archaeol Sci |
| 207 | Stability of canine distemper virus (CDV) after 20 passages in Vero-DST cells expressing the receptor protein for CDV | Non-epidemiological study about CDV sero-prevalence | Vet Microbiol |
| 208 | Saxitoxin increases phocine distemper virus replication upon in-vitro infection in harbor seal immune cells | Non-CDV study | Harmful Algae |
| 209 | Demography and health of “village dogs” in rural Western Uganda | Non-epidemiological study about CDV sero-prevalence | Prev Vet Med |
| 210 | Concomitance and interactions of pathogens in the Iberian wolf (Canis lupus) | Non-domestic dog (Iberian wolf [Canis lupus]) | ResVet Sci |
| 211 | Immunization with plasmid DNA encoding the hemagglutinin and the nucleoprotein confers robust protection against a lethal canine distemper virus challenge | Immunization with CDV | Vaccine |
| 212 | Rabies vaccine is associated with decreased all-cause mortality in dogs | Non-CDV study | Vaccine |
| 213 | Domestic mammals facilitate tick-borne pathogen transmission networks in South African wildlife | Non-CDV study | Biol Cons |
| 214 | Efficient generation of vesicular stomatitis virus (VSV)-pseudotypes bearing morbilliviral glycoproteins and their use in quantifying virus neutralising antibodies | Non-CDV study | Vaccine |
| 215 | Canine distemper of vaccine origin in European mink, Mustela lutreola-a case report | Non-domestic dog | Vet Microbiol |
| 216 | Evolution of Canine Parvovirus in Argentina between years 2003 and 2010: CPV2c has become the predominant variant affecting the domestic dog population | Non-CDV study | Virus Res |
| 217 | A recombinant pseudorabies virus expressing rabies virus glycoprotein: Safety and immunogenicity in dogs | Non-epidemiological study about CDV sero-prevalence | Vaccine |
| 218 | Virological Survey in free-ranging wildcats (Felis silvestris) and feral domestic cats in Portugal | Non-domestic dog (wildcats [Felis silvestris]) | Vet Microbiol |
| 219 | Detection of morbillivirus infection by RT-PCR RFLP analysis in cetaceans and carnivores | Phylogenetic analyses | J Virol Methods |
| 220 | Large-scale assessment of the presence of Darwin’s fox across its newly discovered range | Non-epidemiological study about CDV sero-prevalence | Mamm Biol |
| 221 | Multiplex Amplification Refractory Mutation System PCR (ARMS-PCR) provides sequencing independent typing of canine parvovirus | Non-epidemiological study about CDV sero-prevalence | Infect, Genet Evol |
| 222 | An insulated isothermal PCR method on a field-deployable device for rapid and sensitive detection of canine parvovirus type 2 at points of need | Non-epidemiological study about CDV sero-prevalence | J Virol Methods |
| 223 | Free-ranging domestic dogs (Canis familiaris) as predators and prey in rural Zimbabwe: threats of competition and disease to large wild carnivores | Non-epidemiological study about CDV sero-prevalence | Biol Cons |
| 224 | Detection of non-primate hepaciviruses in UK dogs | Non-CDV study | Virology |
| 225 | Virus recovery from idiopathic conjunctivitis of dogs | Only analyzed idiopathic conjunctivitis | Adv Small Anim Med Surg |
| 226 | Enhanced immunosurveillance for animal morbilliviruses using vesicular stomatitis virus (VSV) pseudotypes | Non-domestic dog (domestic livestock and wild ruminants) | Vaccine |
| 227 | Dietary astaxanthin enhances immune response in dogs | Non-CDV study | Vet Immunol Immunopathol |
| 228 | Is the free-ranging jaguar (Panthera onca) a reservoir for Cytauxzoon felis in Brazil? | Non-domestic dog | Ticks Tick Borne Dis |
| 229 | Vaccination strategies to conserve the endangered African wild dog (Lycaon pictus) | Non-domestic dog (African wild dog [Lycaon pictus]) | Biol Cons |
| 230 | Urban dogs in rural areas: Human-mediated movement defines dog populations in southern Chile | Non-epidemiological study about CDV sero-prevalence | Prev Vet Med |
| 231 | Fine-scale movements of rural free-ranging dogs in conservation areas in the temperate rainforest of the coastal range of southern Chile | Non-epidemiological study about CDV sero-prevalence | Mamm Biol |
| 232 | Phocine distemper virus in the North and European Seas – Data and models, nature and nurture | Non-epidemiological study about CDV sero-prevalence | Biol Cons |
| 233 | Non-suppurative Meningoencephalitis of Unknown Origin in Cats and Dogs: an Immunohistochemical Study | Non-epidemiological study about CDV sero-prevalence | J Comp Pathol |
| 234 | Recombinant canine adenovirus type-2 expressing TgROP16 provides partial protection against acute Toxoplasma gondii infection in mice | Non-CDV study | Infect, Genet Evol |
| 235 | A new multivalent (DHPPi/L4R) canine combination vaccine prevents infection, shedding and clinical signs following experimental challenge with four Leptospira serovars | Non-CDV study | Vaccine |
| 236 | Characterisation of canine parvovirus strains isolated from cats with feline panleukopenia | Non-domestic dog | Res Vet Sci |
| 237 | Contamination of infectious RD-114 virus in vaccines produced using non-feline cell lines | Non-epidemiological study about CDV sero-prevalence | Biologicals |
| 238 | Serosurvey of selected viruses in captive giant pandas (Ailuropoda melanoleuca) in China | Non-domestic dog | Vet Microbiol |
| 239 | Distribution, seasonality and risk factors for tick paralysis in Australian dogs and cats | Non-CDV study | Vet Parasitol |
| 240 | Disease threats to the endangered Iberian lynx (Lynx pardinus) | Non-domestic dog | Vet J |
| 241 | Pathogen exposure in endangered island fox (Urocyon littoralis) populations: Implications for conservation management | Non-domestic dog | Biol Cons |
| 242 | A distinct CDV genotype causing a major epidemic in Alpine wildlife | Non-domestic dog | Vet Microbiol |
| 243 | Changes in blood testosterone concentrations after surgical and chemical sterilization of male free-roaming dogs in southern Chile | Non-CDV study | Theriogenology |
| 244 | Intestinal parasites of dogs on the Galapagos Islands | Non-CDV study | Vet Parasitol |
| 245 | Fatal nocardiosis in a dog caused by multiresistant Nocardia veterana | Non-CDV study | Vet Microbiol |
| 246 | Infectious Hepatopathies in Dogs and Cats | Review | Top Companion Anim Med |
| 247 | Heterologous prime-boost vaccination with a non-replicative vaccinia recombinant vector expressing LACK confers protection against canine visceral leishmaniasis with a predominant Th1-specific immune response | Vaccine against CDV | Vaccine |
| 248 | Experimental infection of dogs (Canis familiaris) with sporulated oocysts of Neospora caninum | Non-epidemiological study about CDV sero-prevalence | Vet Parasitol |
| 249 | Development of a SYBR Green based real-time PCR assay for detection and quantitation of canine parvovirus in faecal samples | Non-epidemiological study about CDV sero-prevalence | J Virol Methods |
| 250 | High resolution melting curve analysis as a new tool for rapid identification of canine parvovirus type 2 strains | Non-epidemiological study about CDV sero-prevalence | Mol Cell Probes |
| 251 | Prevalence of dirofilarial infection in raccoon dogs in Japan | Non-CDV study | Parasitol Int |
| 252 | Bone-marrow changes in infectious diseases and lymphohaemopoietic neoplasias in dogs and cats-a retrospective study | Non-CDV study | J Comp Pathol |
| 253 | The safety and efficacy of the oral rabies vaccine SAG2 in Indian stray dogs | Vaccine study | Vaccine |
| 254 | Efficacy of a multivalent DAPPi-Lmulti canine vaccine against mortality, clinical signs, infection, bacterial excretion, renal carriage and renal lesions caused by Leptospira experimental challenges | Non-CDV study | Vaccine Reports |
| 255 | Vaccine use and disease prevalence in dogs and cats | Vaccine study | Vet Microbiol |
| 256 | Pulmonary Blastomycosis in a Domestic Ferret (Mustela putorius furo) | Non-CDV study | J Exot Pet Medic |
| 257 | Safety trial using the Leishmune® vaccine against canine visceral leishmaniasis in Brazil | Non-CDV study | Vaccine |
| 258 | Shedding of Neospora caninum oocysts by dogs fed different tissues from naturally infected cattle | Non-CDV study | Vet Parasitol |
| 259 | Acute onset of encephalomyelitis with atypical lesions associated with dual infection of Sarcocystis neurona and Toxoplasma gondii in a dog | Non-CDV study | Vet Parasitol |
| 260 | Vegetal extracts influence in vitro on the cell-mediated immunity in carnivores depending on health status, target species and plant taxonomy | Non-CDV study | Industrial Crops and Products |
| 261 | Morbillivirus infections, with special emphasis on morbilliviruses of carnivores | Review | Vet Microbiol |
| 262 | Dietary lutein stimulates immune response in the canine | Non-CDV study | Vet Immunol Immunopathol |
| 263 | Multi-host pathogens and carnivore management in southern Africa | Non-domestic dog | Comp Immunol, Microbiol Infect Dis |
| 264 | Faecal virome of red foxes from peri-urban areas | Non-domestic dog | Comp Immunol, Microbiol Infect Dis |
| 265 | An integrated disease management strategy for the control of rabies in Ethiopian wolves | Non-CDV study | Biol Cons |
| 266 | Expression of 3β-hydroxysteroid dehydrogenase in ovarian and uterine tissue during diestrus and open cervix cystic endometrial hyperplasia-pyometra in the bitch | Non-CDV study | Theriogenology |
| 267 | Experimental infection of European red foxes (Vulpes vulpes) with canine herpesvirus | Non-domestic dog | Veterinary Microbiology |
| 268 | A novel dynamic flow immunochromatographic test (DFICT) using gold nanoparticles for the serological detection of Toxoplasma gondii infection in dogs and cats | Non-CDV study | Biosens Bioelectron |
| 269 | Vaccination with plasmid DNA encoding KMPII, TRYP, LACK and GP63 does not protect dogs against Leishmania infantum experimental challenge | Non-CDV study | Vaccine |
| 270 | Effective immunotherapy against canine visceral leishmaniasis with the FML-vaccine | Non-CDV study | Vaccine |
| 271 | A prime/boost DNA/Modified vaccinia virus Ankara vaccine expressing recombinant Leishmania DNA encoding TRYP is safe and immunogenic in outbred dogs, the reservoir of zoonotic visceral leishmaniasis | Non-CDV study | Vaccine |
| 272 | Suitability of canine herpesvirus as a vector for oral bait vaccination of foxes | Non-CDV study | Vet Microbiol |
| 273 | Multivariate analysis of the immune response to a vaccine as an alternative to the repetition of animal challenge studies for vaccines with demonstrated efficacy | Non-CDV study | Vet Immunol Immunopathol |
| 274 | Introduced species: domestic mammals are more significant transmitters of parasites to native mammals than are feral mammals | Non-epidemiological study about CDV sero-prevalence | Int J Parasitol |
| 275 | Canine and feline pregnancy loss due to viral and non-infectious causes: A review | Review | Theriogenology |
| 276 | Immunotherapy against experimental canine visceral leishmaniasis with the saponin enriched-Leishmune® vaccine | Non-epidemiological study about CDV sero-prevalence | Vaccine |
| 277 | The antiviral activity of six South African plants traditionally used against infections in ethnoveterinary medicine | antiviral activity | Vet Microbiol |
| 278 | First report of Angiostrongylus vasorum and Hepatozoon from a red fox (Vulpes vulpes) from West Virginia, USA | Non-domestic dog/Non-CDV study | Vet Parasitol |
| 279 | Development of canine herpesvirus based antifertility vaccines for foxes using bacterial artificial chromosomes | Non-CDV study | Vaccine |
| 280 | A standardized gating technique for the generation of flow cytometry data for normal canine and normal feline blood lymphocytes | Non-CDV study | Vet Immunol Immunopathol |
| 281 | Canine visceral leishmaniasis: Relationships between clinical status, humoral immune response, haematology and Lutzomyia (Lutzomyia) longipalpis infectivity | Non-CDV study | Vet J |
| 282 | Systemic Infection Due to Candida parapsilosis in a Domestic Ferret (Mustela putorius furo) | Non-domestic dog/Non-CDV study | J Exot Pet Med |
| 283 | Clinical Sarcocystis neurona, Sarcocystis canis, Toxoplasma gondii, and Neospora caninum infections in dogs | Non-domestic dog/Non-CDV study | Vet Parasitol |
| 284 | Age-related alterations to immune parameters in Labrador retriever dogs | Non-epidemiological study about CDV sero-prevalence | Vet Immunol Immunopathol |
| 285 | Biochemical serum profiles in dogs experimentally infected with Angiostrongylus vasorum (Baillet, 1866) | Non-CDV study | Vet Parasitol |
| 286 | The role of veterinary epidemiology in the study of free-roaming dogs and cats | Review | Prev Vet Med |
| 287 | Dog invasion in agroforests: The importance of households, roads and dog population size in the surroundings | Non-epidemiological study about CDV sero-prevalence | Perspectives in Ecology and Conservation |
| 288 | Heterologous antibodies to evaluate the kinetics of the humoral immune response in dogs experimentally infected with Toxoplasma gondii RH strain | Non-CDV study | Vet Parasitol |
| 289 | Molecular epidemiological study of Arctic rabies virus isolates from Greenland and comparison with isolates from throughout the Arctic and Baltic regions | Non-CDV study | Virus Res |
| 290 | Protective vaccination against experimental canine visceral leishmaniasis using a combination of DNA and protein immunization with cysteine proteinases type I and II of L. infantum | Non-CDV study | Vaccine |
| 291 | Companion animal disease surveillance: A new solution to an old problem? | Disease surveillance system for dog | Spatial and Spatio-temporal Epidemiology |
| 292 | Evaluation of a rapid immunodiagnostic test kit for rabies virus | Non-CDV study | J Virol Methods |
| 293 | Prevalence of antibodies against feline panleukopenia virus in client-owned cats in Southern Germany | Non-domestic dog/Non-CDV study | Vet J |
| 294 | Acute toxoplasmosis in three wild arctic foxes (Alopex lagopus) from Svalbard; one with co-infections of Salmonella Enteritidis PT1 and Yersinia pseudotuberculosis serotype 2b | Non-domestic dog/Non-CDV study | Res Vet Sci |
| 295 | Reconciling surveillance systems with limited resources: an evaluation of passive surveillance for rabies in an endemic setting | Non-domestic dog/Non-CDV study | Prev Vet Med |
| 296 | The Potential of Duck Hepatitis Virus (DHV-1) Stimulating the Body Weight Gain and the Effects of Silymarin on It in Duckling | Non-domestic dog/Non-CDV study | Agricultural Sciences in China |
| 297 | Sequence analysis of the phosphoprotein gene of peste des petits ruminants (PPR) virus: editing of the gene transcript | Non-domestic dog/Non-CDV study | Virus Res |
| 298 | Development of vaccination strategies for the management of rabies in African wild dogs | Non-domestic dog/Non-CDV study | Biol Cons |
| 299 | Antibiotic resistance free plasmid DNA expressing LACK protein leads towards a protective Th1 response against Leishmania infantum infection | Non-domestic dog/Non-CDV study | Vaccine |
| 300 | Cloning and expression of mink (Neovison vison) interferon-γ gene and development of an antiviral assay | Non-domestic dog/Non-CDV study | Res Vet Sci |
| 301 | Congenital Peripheral Vestibular Syndrome in a Domestic Ferret (Mustela putorius furo) | Non-domestic dog/Non-CDV study | J Exot Pet Med |
| 302 | Effects of organohalogen pollutants on haematological and urine clinical–chemical parameters in Greenland sledge dogs (Canis familiaris) | Non-CDV study | Ecotox Environ Safe |
| 303 | Development and validation of a real-time PCR assay for specific and sensitive detection of canid herpesvirus 1 | Non-CDV study | J Virol Methods |
| 304 | Canine Necrotizing Encephalitis Associated with Anti-glomerular Basement Membrane Glomerulonephritis | Non-CDV study | J Comp Pathol |
| 305 | Veterinary Vaccines and Their Importance to Animal Health and Public Health | Vaccine against CDV | Procedia in Vaccinology |
| 306 | Aberrant Ancylostoma sp. in the brain of a dog | Non-domestic dog/Non-CDV study | Vet Parasitol |
| 307 | Acute Hendra virus infection: Analysis of the pathogenesis and passive antibody protection in the hamster model | Non-domestic dog/Non-CDV study | Virology |
| 308 | Examining dog owners’ beliefs regarding rabies vaccination during government-funded vaccine clinics in Grenada to improve vaccine coverage rates | Non-CDV study | Prev Vet Med |
| 309 | Molecular epidemiology of rabies in bat-eared foxes (Otocyon megalotis) in South Africa | Non-CDV study | Virus Res |
| 310 | Vaccination of cats with an attenuated recombinant myxoma virus expressing feline calicivirus capsid protein | Non-domestic dog/Non-CDV study | Vaccine |
| 311 | Experimental inoculation of European red foxes with recombinant vaccinia virus expressing zona pellucida C proteins | Non-CDV study | Vaccine |
| 312 | Coronavirus infection of spotted hyenas in the Serengeti ecosystem | Non-domestic dog/Non-CDV study | Vet Microbiol |
| 313 | Development and registration of recombinant veterinary vaccines: The example of the canarypox vector platform | Vaccine against CDV | Vaccine |
| 314 | Extreme Tetralogy of Fallot With Polycythemia in a Ferret (Mustela putorius furo) | Non-domestic dog/Non-CDV study | Top Companion Anim Med |
| 315 | Conflicts between Amur (Siberian) tigers and humans in the Russian Far East | Non-domestic dog/Non-CDV study | Biol Cons |
| 316 | Replication and expression of a swinepox virus vector delivering feline leukemia virus Gag and Env to cell lines of swine and feline origin | Non-domestic dog/Non-CDV study | Virus Res |
| 317 | Isolation and Phylogenetic Characterization of Streptococcus halichoeri from a European Badger (Meles meles) with Pyogranulomatous Pleuropneumonia | Non-domestic dog/Non-CDV study | J Comp Pathol |
| 318 | First evidence of hemoplasma infection in free-ranging Namibian cheetahs (Acinonyx jubatus) | Non-domestic dog/Non-CDV study | Vet Microbiol |
| 319 | Infection of bovine dendritic cells by rinderpest or measles viruses induces different changes in host transcription | Non-domestic dog/Non-CDV study | Virology |
| 320 | Immunophenotyping of immune cell populations in the raccoon (Procyon lotor) | Non-domestic dog/Non-CDV study | Vet Immunol Immunopathol |
| 321 | FIV diversity: FIVPle subtype composition may influence disease outcome in African lions | Non-domestic dog/Non-CDV study | Vet Immunol Immunopathol |
| 322 | Oral vaccination and protection of striped skunks (Mephitis mephitis) against rabies using ONRAB® | Non-domestic dog/Non-CDV study | Vaccine |
| 323 | Non-surgical methods of contraception and sterilization | Non-domestic dog/Non-CDV study | Theriogenology |
| 324 | Feasibility and efficacy of oral rabies vaccine SAG2 in endangered Ethiopian wolves | Non-domestic dog/Non-CDV study | Vaccine |
| 325 | Pyogenic Ventriculitis and Ventricular Empyema associated with Staphylococcus pseudintermedius in a Puppy | Non-CDV study | J Comp Pathol |
| 326 | Poxvirus-vectored vaccines for rabies—A review | Review | Vaccine |
| 327 | Skin diseases of ferrets | Non-domestic dog/Non-CDV study | Semin Avian Exot Pet Med |
| 328 | Neonatal immunity and immunisation in early age: lessons from veterinary medicine | Non-domestic dog/Non-CDV study | Vaccine |
| 329 | Humoral immune response to oral rabies vaccination in raccoon kits: Problems and implications | Non-domestic dog/Non-CDV study | Vaccine |
| 330 | Infectious causes of embryonic and fetal mortality | Non-domestic dog/Non-CDV study | Theriogenology |
| 331 | Anthropogenic environmental change and the emergence of infectious diseases in wildlife | Review | Acta Trop |
| 332 | A Potentially Fatal Mix of Herpes in Zoos | Non-CDV study | Curr Biol |
| 333 | Advances in peste des petits ruminants vaccines | Non-domestic dog/Non-CDV study | Vet Microbiol |
| 334 | Gastric Neuroendocrine Carcinoma Associated with Atrophic Gastritis in the Norwegian Lundehund | Non-domestic dog/Non-CDV study | J Comp Pathol |
| 335 | Plagues and adaptation: Lessons from the Felidae models for SARS and AIDS | Non-domestic dog/Non-CDV study | Biol Cons |
| 336 | Acute fatal sarcocystosis hepatitis in an Indo-Pacific bottlenose dolphin (Tursiops aduncus) in Hong Kong | Non-domestic dog/Non-CDV study | Vet Parasitol |
| 337 | Emerging Ferret Diseases | Non-domestic dog/Non-CDV study | Journal of Exotic Pet Medicine |
| 338 | Rinderpest: The Disease and Its Impact on Humans and Animals | Non-CDV study | Adv Virus Res |
| 339 | Wildlife disease ecology: from theory to policy | Non-epidemiological study about CDV sero-prevalence | Trends Ecol Evol |
| 340 | Reassessing conflicting evolutionary histories of the Paramyxoviridae and the origins of respiroviruses with Bayesian multigene phylogenies | Non-epidemiological study about CDV sero-prevalence | Infect, Genet Evol |
| 341 | Rhinitis and disseminated disease in a ferret (Mustela putorius furo) naturally infected with Sarcocystis neurona | Non-domestic dog/Non-CDV study | Vet Parasitol |
| 342 | Intrathoracic Myxosarcoma in a Dog | Non-domestic dog/Non-CDV study | J Comp Pathol |
| 343 | Antibodies reactive with Ehrlichia canis, Ehrlichia phagocytophila genogroup antigens and the spotted fever group rickettsial antigens, in free-ranging jackals (Canis aureus syriacus) from Israel | Non-domestic dog/Non-CDV study | Vet Parasitol |
| 344 | Meningoencephalitis Associated with Sarcocystis spp. in a Free-Living Japanese Raccoon Dog (Nyctereutes procyonoides viverrinus) | Non-domestic dog/Non-CDV study | J Comp Pathol |
| 345 | Endogenous Lipid Pneumonia in a Ferret (Mustela putorius furo) | Non-domestic dog/Non-CDV study | Journal of Exotic Pet Medicine |
| 346 | Animal behaviour and its role in carnivore conservation: examples of seven deadly threats | Non-CDV study | Animal Behaviour |
| 347 | Development of a hyena immunology toolbox | Non-domestic dog/Non-CDV study | Vet Immunol Immunopathol |
| 348 | Apoptosis in the mammalian CNS: Lessons from animal models | Non-domestic dog/Non-CDV study | Vet J |
| 349 | Polar Bear Encephalitis: Establishment of a Comprehensive Next-generation Pathogen Analysis Pipeline for Captive and Free-living Wildlife | Non-domestic dog/Non-CDV study | Disease in wildlife or exotic species |
| 350 | Factors affecting seroprevalence of Toxoplasma gondii in the endangered Iberian lynx (Lynx pardinus) | Non-domestic dog/Non-CDV study | Vet Parasitol |
| 351 | Behavioural influences on disease risk: implications for conservation and management | Non-domestic dog/Non-CDV study | Animal Behaviour |
| 352 | Kinetics of humoral immune response after rabies VR-G oral vaccination of captive fox cubs (Vulpes vulpes) with or without maternally derived antibodies against the vaccine | Non-domestic dog/Non-CDV study | Vaccine |
| 353 | Ferrets: Examination and Preventive Medicine | Non-domestic dog/Non-CDV study | Vet Clin North Am Exotic Anim Pract |
| 354 | Epidemiology and elimination of rabies in Western Europe | Non-domestic dog/Non-CDV study | Vet J |
| 355 | Effectiveness of an immunocastration vaccine formulation to reduce the gonadal function in female and male mice by Th1/Th2 immune response | Non-domestic dog/Non-CDV study | Theriogenology |
| 356 | Molecular detection and characterization of potentially new Babesia and Theileria species/variants in wild felids from Kenya | Non-domestic dog/Non-CDV study | Acta Trop |
| 357 | Veterinary public health: Past success, new opportunities | Non-domestic dog/Non-CDV study | Prev Vet Med |
| 358 | Human North American River Otter(Lontra canadensis) Attack | Non-domestic dog/Non-CDV study | Wilderness Environ Med |
| 359 | Alternative methods and strategies to reduce, refine, and replace animal use for veterinary vaccine post-licensing safety testing: state of the science and future directions | Non-domestic dog/Non-CDV study | Procedia in Vaccinology |
| 360 | Seroprevalence of Toxoplasma gondii and Neospora caninum in captive maned wolves (Chrysocyon brachyurus) from southeastern and midwestern regions of Brazil | Non-domestic dog/Non-CDV study | Vet Parasitol |
| 361 | Immunization of Male Mice with a New Recombinant GnRH Fusion Protein Reduces the Testicular Function | Non-CDV study | Agricultural Sciences in China |
| 362 | The effects of surgical and chemical castration on intermale aggression, sexual behaviour and play behaviour in the male ferret (Mustela putorius furo) | Non-domestic dog/Non-CDV study | Appl Anim Behav Sci |
| 363 | Detection of multiple Mycoplasma species in bulk tank milk samples using real-time PCR and conventional culture and comparison of test sensitivities | Non-domestic dog/Non-CDV study | J Dairy Science |
| 364 | Population dynamics of infectious diseases: A discrete time model | Non-domestic dog/Non-CDV study | Ecological Modelling |
| 365 | Review of animal models designed to predict the potential allergenicity of novel proteins in genetically modified crops | Review/ Non-domestic dog/Non-CDV study | Regul Toxicol Pharmacol |
| **SCIELO** | | | |
| 366 | Detection and genetic characterization of Mamastrovirus 5 from Brazilian dogs | Non-CDV study | Braz J Microbiol |
| 367 | Detection of respiratory viruses in shelter dogs maintained under varying environmental conditions | Does not mention whey status of vaccination against CDV | Braz J Microbiol |
| 368 | Severe canine distemper outbreak in unvaccinated dogs in Mozambique | Pooled data on CDV outbreak | J South African Vet Assoc |
| 369 | Molecular detection and phylogenetic relationship of wild-type strains of canine distemper virus in symptomatic dogs from Uberlândia, Minas Gerais | Reduced sample size | Arq Brasil Med Vet Zootec |
| 370 | Cytological alterations of the bone marrow and peripheral blood of dogs with canine distemper | Only positive CDV samples | Arq Brasil Med Vet Zootec |
| 371 | Microscopic analysis of the left ventricular myocardium in positive serum dogs to distemper disease | Only positive CDV samples | Pesqui Vet Brasil |
| 372 | Nocardiosis: an overview and additional report of 28 cases in cattle and dogs | Non-CDV study | Rev Instit Med Trop São Paulo |
| 373 | Detection of canine distemper virus by RT-PCR using oligonucleotides targeted to the phosphoprotein, hemagglutinin and neuraminidase genes | RT-PCR Optimization | Arq Brasil Med Vet Zootec |
| 374 | Virus isolation and molecular characterization of canine distemper virus by RT–PCR from a mature dog with multifocal encephalomyelit | Non-epidemiological study about CDV sero-prevalence | Braz J Microbiol |
| 375 | Molecular analysis of the N gene of canine distemper virus in dogs in Brazil | Non-epidemiological study about CDV sero-prevalence | Arq Bras Med Vet Zootec |
| 376 | Evaluation of the urine and leucocytes as biological samples for ante mortem detection of canine distemper virus by RT-PCR assay in naturally infected dogs |  | Arq Bras Med Vet Zootec |
| 377 | Toxoplasma gondii genotyping in a dog co-infected with distemper virus and ehrlichiosis rickettsia | Non-CDV study | Rev Instit Med Trop São Paulo |
| 378 | Restriction pattern of a hemagglutinin gene amplified by RT-PCR from vaccine strains and wild-type canine distemper virus | Only positive CDV samples | Arq Bras Med Vet Zootec |
| 379 | Morphometric analysis of the thymus of puppies infected with the Snyder Hill Strain of canine distemper virus | Non-epidemiological study about CDV sero-prevalence | Arq Brasil Med Vet Zootec |
| 380 | Histopathological lesions in the central nervous system of dogs with encephalitis and molecular diagnosis of canine distemper virus infection | Non-epidemiological study about CDV sero-prevalence | Arq Bras Med Vet Zootec |
| 381 | Simultaneous canine distemper encephalitis and canine parvovirus infection with distemper-associated cardiac necrosis in a pup | Non-epidemiological study about CDV sero-prevalence | Ciênc Rural |
| 382 | Occurrence of apoptosis in leukocytes in the peripheral blood smear and in syncytia in canine distemper infection in vivo | Non-epidemiological study about CDV sero-prevalence | Arq Bras Med Vet Zootec |
| 383 | Detection of canine distemper virus nucleoprotein gene by RT-PCR in urine of dogs with distemper clinical signs |  | Arq Bras Med Vet Zootec |
| 384 | Evaluation of the urine and leucocytes as biological samples for ante mortem detection of canine distemper virus by RT-PCR assay in naturally infected dogs |  | Arq Bras Med Vet Zootec |
| 385 | Antibodies levels against canine distemper virus and canine parvovirus in vaccinated and unvaccinated dogs |  | Arq Bras Med Vet Zootec |
| **Additional references** | | | |
| 386 | Molecular characterization of canine distemper virus from Tamil Nadu, India |  | Indian J Animal Sc |
| 387 | Comparision of the immunofluorescence assay with RT-PCR and Nested PCR in the diagnosis of canine distemper | Study with a sample size <50 | Vet Res Commun |
| 388 | Antibody Prevalence to Canine Distemper Virus (CDV) in Stray Dogs in Turkey |  | Revue Méd Vét |
| 389 | Molecular and Serological Detection of Canine Distemper Virus (CDV) in Rural Dogs, Iran | Study with a sample size <50 | Iranian J Virol |
| 390 | Molecular surveillance of canine distemper virus in diarrhoetic puppies in northeast China from May 2014 to April 2015 |  | J Vet Med Sci |
| 391 | Canine distemper virus: detection of viral RNA by Nested RT-PCR in dogs with clinical diagnosis |  | Braz J Vet Res Anim Sci |
| 392 | Detection of Local Isolates of Canine Distemper Virus by Reverse-Transcription Polymerase Chain Reaction | A study primarily aimed at viral isolation | Int J Curr Microbiol App Sci |
| 393 | Restricted expression of viral surface proteins in canine distemper encephalitis | Dog samples known to be positive for CDV | Acta Neuropathol |
| 394 | Accuracy of a point-of-care ELISA test kit for predicting the presence of protective canine parvovirus and canine distemper virus antibody concentrations in dogs | Pooled results about CDV infection | The Vet J |
| 395 | Detection of IgM antibodies against canine distemper virus in dog and mink sera employing enzyme-linked immunosorbent assay (ELISA) | Pooled results about CDV infection | J Vet Diagn Invest |
| 396 | Evaluation of ELISA based on the conserved and functional middle region of nucleocapsid protein to detect distemper infection in dogs | Pooled results about CDV infection | Vet Microbiol |
| 397 | Development of recombinant nucleocapsid protein based IgM-ELISA for the early detection of  distemper infection in dogs | Pooled results about CDV infection | Vet Immunol Immunopat |
| 398 | Epidemiology and clinical presentation of canine distemper disease in dogs and ferrets in Australia, 2006–2014 | Pooled results about CDV infection | Australian Vet J |
| 399 | Infectious Diseases of Dogs and Cats on Isabela Island, Galapagos |  | J Vet Intern Med |
| 400 | Disease as a threat to endangered species: Ethiopian wolves, domestic dogs and canine pathogens | Pooled results about CDV infection | Anim Cons |
| 401 | Seroprevalence of pathogens in domestic carnivores on the border of Madidi National Park, Bolivia | Reduced sample size | Anim Cons |
| 402 | Surveillance System for Infectious Diseases of Pets, Santiago, Chile | Pilot surveillance system of pet infectious diseases in Santiago. No information for CDV diagnostic | Emerg Infect Dis |
| 403 | Identification of a genetic variant of canine distemper virus from clinical cases in two vaccinated dogs in Mexico | Non-epidemiological study about CDV sero-prevalence | Vet J |
| 404 | Seropositivity of Canine Distemper Virus (CDV) in Dogs Presenting at Abeokuta, Nigeria | High proportion of animals vaccinated against CDV | Public Health Res |
| 405 | Natural Distemper in Vaccinated and Unvaccinated Dogs in Warsaw |  | J Vet Med |
| 406 | Comparison of tissue and fluid samples for the early detection of canine distemper virus in experimentally | Non-epidemiological study about CDV sero-prevalence | Intern Med |
| 407 | Serosurvey of canine distemper virus and canine parvovirus in wild canids and domestic dogs at the rural interface in the Coquimbo Region, Chile | Data merged into another study | Eur J Wildl Res |
| 408 | An immunochromatography assay for rapid antemortem diagnosis of dogs suspected to have canine distemper |  | J Virol Methods |
| 409 | Detection and differentiation of field and vaccine strains of canine distemper virus using reverse transcription followed by nested real time PCR (RT-nqPCR) and RFLP analysis | Probable sample overlap | J Virol Methods |
| **410** | **Canine distemper virus detection in asymptomatic and non vaccinated dogs** | **Reduced sample size** | **Pesq Vet Bras** |
| 411 | Prevalence of canine infectious respiratory pathogens in asymptomatic dogs presented at US animal shelters |  | J Small Anim Pract |
| 412 | Molecular surveillance of traditional and emerging pathogens associated with canine infectious respiratory disease |  | Vet Microbiol |
| 413 | Etiologic Study of Upper Respiratory Infections of Household Dogs | Primarily focused on viral isolation | J Vet Med Sci |
| 414 | Isolation of canine distemper viruses from domestic dogs in South Africa using Vero.DogSLAM cells and its application to diagnosis | Primarily focused on viral isolation | Afr J Microbiol Res |
| 415 | Detection of canine distemper virus nucleoprotein RNA by reverse transcription-PCR using serum, whole blood, and cerebrospinal fluid from dogs with distemper | Reduced sample size | J Clin Microbiol |
| 416 | Comparative evaluation of clinical samples from naturally infected dogs for early detection of canine distemper virus |  | Turk J Vet Anim Sci |
| 417 | Presence of infectious agents and co-infections in diarrheic dogs determined with a real-time polymerase chain reaction-based panel |  | BMC Vet Res |
| 418 | Detection of canine distemper virus by reverse transcriptase-polymerase chain reaction in the urine of dogs with clinical signs of distemper encephalitis |  | Res Vet Science |
| 419 | Comparison of one-step RT-PCR and a nested PCR for the detection of canine distemper virus in clinical samples | Reduced sample size | Aust Vet J |
| 420 | Detection of canine distemper virus (CDV) through one step RT-PCR combined with nested PCR |  | J Vet Sci |
| 421 | Soroprevalência das infecções por parvovírus, adenovírus, coronavírus canino e pelo vírus da cinomose em cães de Santa Maria, Rio Grande do Sul, Brasil |  | Ciênc Rural |
| 422 | Characteristics of a Canine Distemper Virus Outbreak in Dichato, Chile Following the February 2010 Earthquake |  | Animals |
| 423 | Wild canids, domestic dogs and their pathogens in Southeast Brazil: disease threats for canid conservation |  | Biodivers Conserv |
| 424 | Epidemiology of Canine distemper and Canine parvovirus in pet dogs in Wenzhou, China |  | Indian J Anim Res |
| 425 | Phylogenetic analysis of canine distemper viruses isolated from vaccinated dogs in Wuhan | Reduced sample size | The J Vet Med Sci |
| 426 | Evaluation of a Direct Immunofluorescent Assay and/or Conjunctival Cytology for Detection of Canine Distemper Virus Antigen |  | Viral Immunol |
| 427 | Genotyping and pathogenic characterization of canine distemper virus based on mutations in the hemagglutinin gene in Chinese domestic dogs |  | Pol J Vet Sci |
| 428 | Update on canine distemper virus (CDV) strains of Arctic-like lineage detected in dogs in Italy |  | Vet Ital |
| 429 | Detection by hemi-nested reverse transcription polymerase chain reaction and genetic characterization of wild type strains of Canine distemper virus in suspected infected dogs |  | J Vet Diagn Invest |
| 430 | Evaluation of RT-PCR and hemi-nested RT-PCR in brain samples from dogs with neurologic signs compatible with distemper | Diagnosis post mortem | Braz J Vet Res Anim Sci |
| 431 | Detection and differentiation of wild-type and vaccine strains of canine distemper virus by a duplex reverse transcription polymerase chain reaction |  | Iranian J Vet Res |
| 432 | Canine distemper virus detection in asymptomatic and non vaccinated dogs | Reduced sample size | Pesq Vet Bras |
| 433 | Assessment of canine distemper virus infection in vaccinated and unvaccinated dogs |  | Indian J Biotech |
| 434 | A multiplex reverse transcription-nested polymerase chain reaction for detection and differentiation of wild-type and vaccine strains of canine distemper virus | Reduced sample size | Virology J |
| 435 | Analysis of infection epidemiological distemper virus, dogs in the municipality of Garanhuns, Pernambuco, Brazil |  | Semina: Ciênc Agrár |
| 436 | Detection of Canine Distemper Virus in Blood Samples by Reverse Transcription Loop-Mediated Isothermal Amplification |  | J Vet Med |
| 437 | Prevalence of respiratory viruses isolated from dogs in Thailand during 2008-2009 |  | Asian Biomed |
| 438 | Prevalence of Canine Distemper Virus in Dogs in Northern Plateau State, Nigeria |  | Saudi J Medicine |
| 439 | Molecular detection, epidemiological analysis, and risk factors associated with infection by canine distemper virus in Recife, Pernambuco |  | Med Vet |
