# Supplementary material for: Molecular and serological surveys of canine distemper virus: A meta-analysis of cross-sectional studies
Source: PLoS One. 2019 May 29;14(5):e0217594. doi: 10.1371/journal.pone.0217594 (PMC6541297; doi:10.1371/journal.pone.0217594)
Supplement: S2 Table — (DOCX) [file pone.0217594.s005.docx]

**S2 Table. The characteristics of the studies included in the meta-analysis**

|  | **ID^1^** | **Biological material collected** | **Total dogs/specimens** | **(%) CDV’s positivity** | **Diagnostic method** | **Gene (molecular detection)** | **Suspected to have CD?** | **Clinical CD sign** | **CDV vaccine status** | **Evidence of CDV's outbreak?** | **Study period** | **Place of study** |
| --- | --- | --- | --- | --- | --- | --- | --- | --- | --- | --- | --- | --- |
| 1 | An DJ et al 2008. An immunochromatography assay for rapid antemortem diagnosis of dogs suspected to have canine distemper | NFI^2^, CS^3^, BL^4^ | 66 | 43.9 | IC^5^  Nested PCR | NP^6^ gene | Dogs suspected to have CD | F^7^, LA^8^, V^9^, D^10^, DH^11^, EM^12^ | NS^13^ | NS | 2005-2006 | Seoul, South Korea |
| 2 | Castanheira P et al 2014. Molecular and serological surveillance of canine enteric viruses in stray dogs from Vila do Maio, Cape Verde | Rectal swabs/blood samples | 88 | 51 | ELISA (IgG anti-N) | - | Health status | - | Non-vaccinated | - | 2010-2011 | Cape Verde |
| 3 | Alves CDBT et al 2018. Identification of enteric viruses circulating in a dog population with low vaccine coverage | Fecal samples | 77 | 41.6 | Nested PCR | NP gene | Suspected of Canine GaE^14^ | GaE | 7.7% complete vaccination | NS | 2008-2014 | Brazil |
| 4 | Ashmi JM et al 2017. Molecular characterization of canine distemper virus from Tamil Nadu, India | Ocular and nasal swabs | 90 | 23 | Nested PCR | NP gene | CDV suspected dogs | NS | NS | NS | NS | India |
| 5 | Gencay A et al 2004. Antibody prevalence to canine distemper virus (CDV) in stray dogs in Turkey | Serum samples | 609 | 55 | Virus neutralization assay | - | Clinically healthy  stray dogs | - | - | NS | 2000-2002 | Turkey |
| 6 | McRee A et al 2014. Serological detection of infection with canine distemper virus, canine parvovirus and canine adenovirus in communal dogs from Zimbabwe | Blood samples/serum | 225 | 33 | ELISA (IgG) | - | NS | - | Had no vaccination history | NS | 2012 | Zimbabwe |
| 7 | Li C et al 2018. Molecular surveillance of canine distemper virus in diarrhoetic puppies in northeast China from May 2014 to April 2015 | Fecal samples | 201 | 24.88 | Nested PCR | NP gene | Diarrhoetic dogs | D | 44% vaccination in CDV+ | - | 2014-2015 | China |
| 8 | Gebara CMS et al 2004. Detection of canine distemper virus nucleoprotein gene by RT-PCR in urine of dogs with distemper clinical signs | Urine samples | 87 | 47 | RT-PCR | NP gene | Clinical signs suggestive of CD | Systemic and neurological signs | NS | NS | 2001 | Brazil |
| 9 | Negrão FJ et al 2007. Evaluation of the urine and leucocytes as biological samples for ante mortem detection of canine distemper virus by RT-PCR assay in naturally infected dogs | Urine and blood samples | 188 | 60.1 | RT-PCR | NP gene | Clinical suspicion of CD | Systemic and neurological signs | NS | NS | 2004 | Brazil |
| 10 | Alcalde R et al 2013. Canine distemper virus: detection of viral RNA by nested RT-PCR in dogs with clinical diagnosis | Blood, saliva and urine samples | 100 | 44 | Nested PCR | NP gene | Clinically suspected of CD | Respiratory, gastrointestinal, and neurological signs | Not vaccinated | NS | NS | Brazil |
| 11 | Romanutti C et al 2016. RT-PCR and sequence analysis of the full-length fusion protein of Canine Distemper Virus from domestic dogs | Clotted blood samples | 236 | 35.6 | RT-PCR | NP gene | - | NS | 70.2% vaccination in CDV+ | NS | 2007-2014 | Argentina |
| 12 | Calderon MG et al 2007. Detection by RT-PCR and genetic characterization of canine distemper virus from vaccinated and non-vaccinated dogs in Argentina | Clotted blood samples | 99 | 73.7 | RT-PCR | NP gene | - | Respiratory, Gastrointestinal, PuO, NeuS | Vaccination records | NS | 2003-2004 | Argentina |
| 13 | Jin Y et al 2017. Canine distemper viral infection threatens the giant panda population in China | Blood samples | 125 | 72 | ELISA (IgG) | - | - | - | Unvaccinated | Yes | 2013-2015 | China |
| 14 | Fischer CDB et al 2016. Phylogenetic analysis of canine distemper virus in South America clade 1 reveals unique molecular signatures of the local epidemic | Whole blood, urine, conjunctive and rectal swabs | 175 | 50.9 | IC  qPCR | NP gene | CD signs | Systemic, respiratory, gastroenterical and neurological signs | Minority was vaccinated | NS | 2010-2011 | Brazil |
| 15 | Curi NHA et al 2016. Prevalence and risk factors for viral exposure in rural dogs around protected areas of the Atlantic forest | Blood samples/serum | 320 | 15 | Serum neutralization | - | - | - | Animals vaccination were excluded | NS | 2011-2012 | Brazil |
| 16 | Acosta-Jamett G et al 2015. Epidemiology of canine distemper and canine parvovirus in domestic dogs in urban and rural areas of the Araucanía region in Chile | Blood samples/serum | 500 | 52 | ELISA (IgM) | - | - | - | Low vaccination coverage | NS | 2009-2012 | Chile |
| 17 | Fung HL et al 2014. Domestic dog health worsens with socio-economic deprivation of their home communities | Blood and fecal samples | 78 | 61.5 | Serum neutralization | - | - | - | - | NS | 2011 | Panama |
| 18 | Sepúlveda MA et al 2014. Invasive American Mink: linking pathogen risk between domestic and endangered carnivores | Blood samples/serum | 59 | 42 | Serum neutralization | - | - | - | Animals vaccination were excluded | NS | 2010 | Chile |
| 19 | Furtado MM et al 2013. Serosurvey for selected viral infections in free ranging Jaguars (Panthera Onca) and domestic carnivores in Brazilian Cerrado, Pantanal, and amazon | Blood samples/serum | 174 | 41 | Serum neutralization | - | - | - | Low vaccination coverage | NS | 2008-2010 | Brazil |
| 20 | Woodroffe R et al 2012. Contact with domestic dogs increases pathogen exposure in endangered African Wild Dogs (Lycaon pictus) | Blood samples/serum | 184 | 48 | Serum neutralization | - | - | - | Unvaccinated | NS | 2001-2009 | Kenya |
| 21 | Acosta-Jamett G et al 2011. Urban domestic dog populations as a source of canine distemper virus for wild carnivores in the Coquimbo region of Chile | Blood samples/serum | 392 | 57 | Serum neutralization | - | - | - | Serum neutralization | Yes | 2005-2007 | Chile |
| 22 | Gowtage-Sequeira S et al 2009. Epidemiology, pathology, and genetic analysis of a canine distemper epidemic in Namibia | Blood samples/serum | 90 | 37 | Serum neutralization | - | For some, CD signs |  | Unvaccinated | Yes | 2001-2003 | Namibia |
| 23 | Nava AFD et al 2008. First evidence of canine distemper in Brazilian free-ranging Felids | Blood samples/serum | 111 | 40.5 | Serum neutralization | - | - | - | Unvaccinated | NS | 1999-2005 | Brazil |
| 24 | Avizeh R et al 2007. Antibody titers against canine distemper virus in unvaccinated rural dogs from Ahvaz, Iran | Blood samples/serum | 97 | 17.5 | Immunofluorescence (IgG) | - | - | - | Unvaccinated | NS | 2004-2005 | Iran |
| 25 | Diaz NM et al 2016. Dog overpopulation and burden of exposure to canine distemper virus and other pathogens on Santa Cruz Island, Galapagos | Blood samples/plasma | 83 | 36 | ELISA (IgG) | - | - | - | Unvaccinated | - | 2014 | Galapagos |
| 26 | Budaszewski RF et al 2014. Genotyping of canine distemper virus strains circulating in Brazil from 2008 to 2012 | Rectal swabs | 386 | 40.2 | Nested PCR | NP gene | Animals with or without clinical signs suggestive of CD | Respiratory, dermatological, gastrointestinal and NeuS | Low vaccination coverage | NS | 2008-2012 | Brazil |
| 27 | Dowgier G et al 2017. A molecular survey for selected viral enteropathogens revealed a limited role of Canine circovirus in the development of canine acute gastroenteritis | Fecal samples and/or rectal swabs | 219 | 0 | qPCR | NP gene | Canine acute gastroenteritis | - | NS | NS | 2013-2016 | Italy |
| 28 | Hass R et al 2008. Antibodies levels against canine distemper virus and canine parvovirus in vaccinated and unvaccinated dogs | Blood samples/serum | 132 | 58.3 | Serum neutralization | - | - | Respiratory, gastrointestinal and NeuS | Unvaccinated | NS | 2005-2006 | Brazil |
| 29 | Levy JK et al 2008. Infectious diseases of dogs and Cats on Isabela Island, Galapagos | Blood samples/plasma | 95 | 22 | Serum neutralization | - | - | - | Unvaccinated | Yes | 2004 | Galapagos |
| 30 | Jóswik A et al 2002. Natural distemper in vaccinated and unvaccinated dogs in Warsaw | Mucous membrane | 224 | 22 | Immunofluorescence (Ag) | - | Clinical signs suggestive of CD | Systemic, respiratory, gastrointestinal, neurological signs | Low vaccination coverage | NS | 1998-2001 | Poland |
| 31 | Albrechtová K et al 2011. Occurrence of filaria in domestic dogs of Samburu pastoralists in Northern Kenya and its associations with canine distemper | Blood samples/serum | 235 | 18.3 | Immunofluorescence | - | - | - | Unvaccinated | Yes | 2006-2007 | Kenya |
| 32 | Lavan R & Knesl O 2015. Prevalence of canine infectious respiratory pathogens in asymptomatic dogs presented at US animal shelters | Blood samples | 503 | 7.4 | qPCR | - | - | - | Unknown | - | 2011-2012 | United States |
| 33 | Decaro N et al 2016. Molecular surveillance of traditional and emerging pathogens associated with canine infectious respiratory disease | Nasal and/or oropharyngeal swabs | 78 | 0 | qPCR | NP gene | Canine infectious respiratory | - | NS | - | 2011 and 2013 | Italy |
| 34 | Gizzi ABR et al 2014. Presence of infectious agents and co-infections in diarrheic dogs determined with a real-time polymerase chain reaction-based panel | Fecal samples | 104 | 8.7 | qPCR | Phosphoprotein gene | Diarrheic | - | NS | - | - | Brazil |
| 35 | Kim YH et al 2001. Detection of canine distemper virus (CDV) through one step RT-PCR combined with nested PCR | Blood samples | 55 | 88 | RT-PCR  Nested PCR | NP gene | Clinical signs suggestive of CD | Systemic, respiratoryl, gastroenteritis and NeuS | NS | NS | - | South Korea |
| 36 | Dezengrini R et al 2007. Soroprevalência das infecções por parvovírus, adenovírus, coronavírus canino e pelo vírus da cinomose em cães de Santa Maria, Rio Grande do Sul, Brasil | Blood samples/serum | 817 | 27.29 | Serum neutralization | - | - | - | Unvaccinated | NS | 2004-2005 | Brazil |
| 37 | Garde E et al 2013. Characteristics of a Canine Distemper Virus Outbreak in Dichato, Chile Following the February 2010 Earthquake | Blood samples/serum | 104 | 51.9 | ELISA (IgM) | - | Clinical signs suggestive of CD | - | Low vaccination coverage | Yes | 2010 | Chile |
| 38 | Curi NHA et al 2010. Wild canids, domestic dogs and their pathogens in Southeast Brazil: disease threats for canid conservation | Blood samples/serum | 70 | 65.7 | Serum neutralization | - | - | - | Unvaccinated | NS | 2004-2005 | Brazil |
| 39 | Luo H et al 2017. Epidemiology of Canine distemper and Canine parvovirus in pet dogs in Wenzhou, China | Eyes and nasal secretions | 2406 | 28.47 | IC (Ag) | - | - | - | Low vaccination coverage | NS | 2010-2014 | China |
| 40 | Belsare AV et al 2014. Epidemiology of Viral Pathogens of Free-Ranging Dogs and Indian Foxes in a Human-Dominated Landscape in Central India | Blood samples/serum | 146 | 72 | ELISA | - | - | - | Unvaccinated | NS | 2011-2012 | India |
| 41 | Millán J et al 2013. Serosurvey of Dogs for Human, Livestock, and Wildlife Pathogens, Uganda | Blood samples/serum | 92 | 100 | ELISA (IgG) | - | - | - | Unvaccinated | NS | 2011 | Uganda |
| 42 | Athanasiou et al 2017. Evaluation of a Direct Immunofluorescent Assay and/or Conjunctival Cytology for Detection of Canine Distemper Virus Antigen | Ocular swab | 57 | 33.3 | RT-PCR  Immunofluorescence | L gene | Signs compatible with CD | NeuS, myoclonus, respiratory and GaE | Unvaccinated or unknown vaccination history | NS | - | Greece |
| 43 | Chen et al 2018. Genotyping and pathogenic characterization of canine distemper virus based on mutations in the hemagglutinin gene in Chinese domestic dogs | Blood samples | 132 | 43.9 | RT-PCR | NP gene | Clinical signs suggestive of CD | - | NS | NS | 2016-2017 | China |
| 44 | Mira et al 2018. Update on canine distemper virus (CDV) strains of Arctic-like lineage detected in dogs in Italy | CS, NIF and rectal swabs;  or organs (brain, lungs, spleen, kidneys, liver, intestine). | 91 | 36.3 | RT-PCR | P gene | Clinical signs of distemper | Neurological, respiratory, or enteric signs | NS | NS | 2015-2016 | Italy |
| 45 | Di Francesco et al 2012. Detection by hemi-nested reverse transcription polymerase chain reaction and genetic characterization of wild type strains of Canine distemper virus in suspected infected dogs | NIF, genital and rectal swabs, buffy coats, blood sera, cerebrospinal fluid, urine and various organs | 53 | 56.6 | Hemi-nested PCR | NP gene | - | Gastrointestinal, respiratory, and neurological signs | Most were probably vaccinated | NS | 2005-2008 | Italy |
| 46 | Dong et al 2015. Detection and differentiation of wild-type and vaccine strains of canine distemper virus by a duplex reverse transcription polymerase chain reaction | Pooled sample | 67 | 49 | RT-PCR | NP gene | Clinical signs suggestive of CD | NS | NS | NS | NS | China |
| 47 | Latha et al 2007. Assessment of canine distemper virus infection in vaccinated and unvaccinated dogs | CS | 160 | 70 | ELISA | - | Clinical signs  suggestive of CD | Conjunctival and nasal discharge, diarrhoeae, fever, skin pustules, hyperkeratosis and CNS | Informed vaccine status | NS | 2002 | India |
| 48 | Cho et al 2005. Detection of Canine Distemper Virus in Blood Samples by Reverse Transcription Loop-Mediated Isothermal Amplification | Blood sample | 50 | 70 | RT-PCR | NP gene | Clinical signs  suggestive of CD | - | NS | NS | 2003-2004 | South Korea |
| 49 | Lúcio et al 2014. Analysis of infection epidemiological distemper virus, dogs in the municipality of Garanhuns, Pernambuco, Brazil | Blood sample | 104 | 90 | IC | - | - | - | Non vaccinated | NS | 2012 | Brazil |
| 50 | Posuwan et al 2010. Prevalence of respiratory viruses isolated from dogs in Thailand during 2008-2009 | Nasal Swab | 211 | 2.4 | Nested PCR | NP gene | 109 dogs with respiratory disease | Respiratory sign | NS | NS | 2008-2009 | Thailand |
| 51 | Wang et al 2018. A fast and simple one‑step duplex PCR assay for canine distemper virus (CDV) and canine coronavirus (CCoV) detection. | Blood sample | 173 | 20.8 | RT-PCR | H gene | Dogs suspected of having CDV | F, V, coughing, D, NeuS | NS | NS | 2014-2015 | China |
| 52 | Ki et al 2017. Prevalence of Canine Distemper Virus in Dogs in Northern Plateau State, Nigeria | Blood sample | 150 | 45.3 | IC |  | - | - | Few vaccinated animals | NS | NS | Nigeria |
| 53 | Silva et al 2018. Molecular detection, epidemiological analysis, and risk factors associated with infection by canine distemper virus in Recife, Pernambuco | Blood sample | 146 | 27.4 | RT-PCR | NP gene | Dogs suspected of having CDV | - | Few vaccinated animals |  | 2012-2013 | Brazil |

^1^ID: Identification of the study; ^2^NIF: Nasal irrigation fluid; ^3^CS: Conjunctival swab; ^4^BL: Blood lymphocytes. ^5^IC:Immunochromatography; ^6^NP: Nucleoprotein; ^7^F: Fever; ^8^LA: Lack of appetite; ^9^V: Vomiting; ^10^D: Diarrhea; ^11^DH: Dehydration; ^12^EM: Emaciation; ^13^NS: Not specified; ^14^GaE: Gastroenteritis; ^15^PuO: Purulent ocular; ^16^NaD: Nasal discharge; ^17^ToL: Tonsillitis; ^18^PhG: Pharyngitis. ^19^BrT: Bronchitis; ^20^CaP: Catarrhal pneumonia; ^21^NeuS: Neurological signs; ^22^CNS: Central nervous system
