# Supplementary material for: Molecular and serological surveys of canine distemper virus: A meta-analysis of cross-sectional studies
Source: PLoS One. 2019 May 29;14(5):e0217594. doi: 10.1371/journal.pone.0217594 (PMC6541297; doi:10.1371/journal.pone.0217594)
Supplement: S3 Table — (DOCX) [file pone.0217594.s006.docx]

**S3 Table. Frequency of CDV infection regarding the regions of origin of the articles.**

| **ID** | **Region (City, province/state, country)** | **CDV POS (95% CI)** | **Pooled CDV POS (95% CI)** | **N (POS)/N** |
| --- | --- | --- | --- | --- |
| Chen et al 2018 | Beijing, China | 44 (36-52) | 34 (21-47) | 951/3104 |
| Li Chunqiu et al 2018 | Heilongjiang, China | 25 (19-31) |  |  |
| Wang et al 2018 | Beijing, Anhui and  Shanxi, China | 21 (15-27) |  |  |
| Dong et al 2015 | Guangdong, China | 49 (38-61) |  |  |
| Jin et al 2017 | Shaanxi, China | 71 (63-78) |  |  |
| Luo et al 2017 | Wenzhou, Zhejiang, China | 28 (27-30) |  |  |
| Ashmi et al 2017 | Chenaai, Tamil Nadu, India | 23 (16-33) |  | 238/396 |
| Belsare et al 2014 | Great Indian Bustard Sanctuary, India | 72 (64-79) |  |  |
| Latha et al 2007 | Chenaai, Tamil Nadu, India | 70 (62-77) |  |  |
| Dong-Jun et al 2008 | Seoul, South Korea | 44 (33-56) | 69 (40-91) | 109/167 |
| Cho et al 2005 | Gwangju, South Korea | 70 (56-81) |  |  |
| Kim et al 2002 | Seoul, South Korea | 88 (77-94) |  |  |
| Avizeh et al 2007 | Ahvaz, Iran | 18 (11-26) |  | 17/97 |
| Posuwan et al 2010 | Bangkok, Thailand | 3 (1-8) |  | 3/102 |
| Gencay et al 2004 | Ankara, Mugla and Istanbul, Turkey | 9 (7-12) |  | 55/609 |
| Dowgier et al 2017 | Valenzano, Bari, Italy | 0 (0-2) | 14 (0-50) | 63/441 |
| Decaro et al 2016 | Valenzano, Bari, Italy | 0 (0-5) |  |  |
| Mira et al 2018 | Palermo, Italy | 36 (27-47) |  |  |
| Di Francesco et al 2012 | Central region, Italy | 57 (43-69) |  |  |
| Józwik et al 2002 | Warsaw, Poland | 22 (17-28) |  | 50/224 |
| Athanasiou et al 2017 | Greece | 33 (22-46) |  | 19/57 |
| Ki et al 2017 | Plateu State, Nigeria | 45 (38-53) |  | 68/150 |
| Woodroffe et al 2012 | Laikipia, Samburu and Isiolo, Kenya | 48 (41-55) | 30 (26-35) | 131/419 |
| Albrechtová et al 2011 | Kenya | 18 (14-24) |  |  |
| Gowtage-Sequeira et al 2009 | Town Council roundups, Swakopmund, Walvis Bay and Luderitz, Namibia | 37 (27-47) |  | 33/90 |
| Castanheira et al 2014 | Vila do Maio, Maio Island, Cape Verde | 51 (41-61) |  | 45/88 |
| Castanheira et al 2014 | Vila do Maio, Maio Island, Cape Verde | 4 (2-9) |  | 6/146 |
| McRee et al 2014 | Hwange District, Zimbabwe | 33 (27-40) |  | 75/225 |
| Millán et al 2013 | Queen Elizabeth, Bwindi Impenetrable and Mgahinga Gorilla Parks, Uganda | 100 (96-100) |  | 92/92 |
| Romanutti et al 2015 | Buenos Aires, Argentina | 36 (30-42) | 47 (42-52) | 157/335 |
| Calderon et al 2007 | Buenos Aires, Argentina | 74 (64-81) |  |  |
| Diaz et al 2016 | Santa Cruz, Galapagos | 36 (27-47) | 28 (22-35) | 51/178 |
| Levy et al 2008 | Puerto Villamil, Galapagos | 22 (15-31) |  |  |
| Garde et al 2013 | Dichato, Region del Biobío, Chile | 52 (42-61) | 52 (48-57) | 560/1055 |
| Acosta-Jamett et al 2015 | Araucanía region, Chile | 51 (47-56) |  |  |
| Sepúlveda et al 2014 | Chaihuím and Cadillal Alto, Chile | 42 (31-55) |  |  |
| Acosta-Jamett et al 2011 | Coquimbo region, Chile | 57 (52-62) |  |  |
| Alves et al 2018 | Rio Grande do Sul, Santa Catarina, Paraná, Rio de Janeiro, São Paulo, Mato Grosso, Rondônia and Acre, Brazil | 42 (31-53) | 39 (29-50) | 583/1404 |
| Fischer et al 2016 | Canoas, Rio Grande do Sul, Brazil | 51 (44-58) |  |  |
| Silva et al 2018 | Recife, Pernambuco, Brazil | 27 (21-35) |  |  |
| Budaszewski et al 2014 | Rio Grande do Sul, Santa Catarina, Paraná,  São Paulo, Rio de Janeiro, Rondônia and Mato Grosso, Brazil | 40 (35-45) |  |  |
| Gizzi et al 2014 | Curitiba, Paraná, Brazil | 9 (5-16) |  |  |
| Alcalde et al 2013 | São Paulo, Brazil | 44 (35-54) |  |  |
| Negrão et al 2007 | Londrina, Brazil | 66 (59-73) |  |  |
| Gebara et al 2004 | Londrina and Maringá, Brazil | 47 (37-58) |  |  |
| Present study | Jataí, Goiás, Brazil | 34 (27-42) |  |  |
| Curi et al 2016 | Minas Gerais, Brazil | 15 (12-19) | 46 (31-62) | 614/1828 |
| Fischer et al 2016 | Canoas, Rio Grande do Sul, Brazil | 31 (25-39) |  |  |
| Furtado et al 2013 | Emas National and Cantão State Parks, Pantanal, Brazil | 41 (34-48) |  |  |
| Curi et al 2010 | Minas Gerais, Brazil | 66 (54-76) |  |  |
| Nava et al 2008 | São Paulo and Mato Grosso do Sul, Brazil | 41 (32-50) |  |  |
| Lúcio et al 2014 | Garanhuns, Pernambuco, Brazil | 90 (83-95) |  |  |
| Dezengrini et al 2007 | Santa Maria, Rio Grande do Sul, Brazil | 27 (24-30) |  |  |
| Lavan et al 2015 | California, Arizona, Colorado, Florida, Indiana, Pennsylvania, Virginia and South Carolina, USA | 7 (5-10) |  | 37/503 |
| Fung et al 2014 | Lagartera Grande, Las Pavas and Los Hules, Panama | 62 (50-72) |  | 48/78 |
