# Supplementary material for: Molecular and serological surveys of canine distemper virus: A meta-analysis of cross-sectional studies
Source: PLoS One. 2019 May 29;14(5):e0217594. doi: 10.1371/journal.pone.0217594 (PMC6541297; doi:10.1371/journal.pone.0217594)
Supplement: S4 Table — (DOCX) [file pone.0217594.s007.docx]

**S4 Table. The sensitivity analysis to estimate of the frequency of CDV positivity**

| **Study Omitted** | **Frequency (95% CI)** |
| --- | --- |
| **Molecular assays** | |
| Alves et al 2018 | 0.35 (0.27-0.43) |
| Chen et al 2018 | 0.35 (0.27-0.43) |
| Li Chunqiu et al 2018 | 0.36 (0.28-0.44) |
| Mira et al 2018 | 0.35 (0.27-0.43) |
| Silva et al 2018 | 0.35 (0.27-0.43) |
| Wang et al 2018 | 0.36 (0.28-0.44) |
| Ashmi et al 2017 | 0.36 (0.28-0.44) |
| Dowgier et al 2017 | 0.37 (0.28-0.46) |
| Decaro et al 2016 | 0.37 (0.28-0.45) |
| Fischer et al 2016 | 0.35 (0.27-0.42) |
| Dong et al 2015 | 0.35 (0.27-0.43) |
| Lavan et al 2015 | 0.36 (0.28-0.45) |
| Romanutti et al 2015 | 0.35 (0.27-0.43) |
| Budaszewski et al 2014 | 0.35 (0.27-0.43) |
| Castanheira et al 2014 | 0.36 (0.28-0.45) |
| Gizzi et al 2014 | 0.36 (0.28-0.44) |
| Alcalde et al 2013 | 0.35 (0.27-0.43) |
| Di Francesco et al 2012 | 0.34 (0.26-0.42) |
| Posuwan et al 2010 | 0.37 (0.28 -0.45) |
| Dong-Jun et al 2008 | 0.35 (0.27-0.43) |
| Calderon et al 2007 | 0.34 (0.26-0.41) |
| Negrão et al 2007 | 0.34 (0.26-0.41) |
| Cho et al 2005 | 0.34 (0.26-0.42) |
| Gebara et al 2004 | 0.35 (0.27-0.43) |
| Kim et al 2001 | 0.33 (0.26-0.40) |
| Present study | 0.35 (0.27-0.43) |
| Combined | 0.35 (0.27-0.43) |
| **Antibody assays** | |
| Jin et al 2017 | 0.44 (0.28-0.60) |
| Curi et al 2016 | 0.47 (0.31-0.63) |
| Diaz et al 2016 | 0.46 (0.30-0.62) |
| Acosta-Jamett et al 2015 | 0.45 (0.29-0.62) |
| Belsare et al 2014 | 0.44 (0.28-0.60) |
| Castanheira et al 2014 | 0.45 (0.29-0.61) |
| Fung et al 2014 | 0.45 (0.29-0.61) |
| Lúcio et al 2014 | 0.44 (0.28-0.59) |
| McRee et al 2014 | 0.46 (0.30-0.62) |
| Sepúlveda et al 2014 | 0.46 (0.30-0.62) |
| Furtado et al 2013 | 0.46 (0.30-0.62) |
| Garde et al 2013 | 0.45 (0.29-0.61) |
| Millán et al 2013 | 0.43 (0.33-0.53) |
| Woodroffe et al 2012 | 0.45 (0.29-0.62) |
| Acosta-Jamett et al 2011 | 0.45 (0.29-0.61) |
| Albrechtová et al 2011 | 0.47 (0.31-0.63) |
| Curi et al 2010 | 0.45 (0.29-0.61) |
| Gowtage-Sequeira et al 2009 | 0.46 (0.30-0.62) |
| Levy et al 2008 | 0.47 (0.31-0.63) |
| Nava et al 2008 | 0.46 (0.30-0.62) |
| Avizeh et al 2007 | 0.47 (0.31-0.63) |
| Dezengrini et al 2007 | 0.46 (0.30-0.63) |
| Gencay et al 2004 | 0.47 (0.33-0.62) |
| Combined | 0.46 (0.30-0.61) |
| **Antigen assays** | |
| Athanasiou et al 2017 | 0.39 (0.25-0.53) |
| Ki et al 2017 | 0.35 (0.21-0.49) |
| Luo et al 2017 | 0.39 (0.21-0.57) |
| Fischer et al 2016 | 0.38 (0.23-0.53) |
| Latha et al 2007 | 0.30 (0.24-0.36) |
| Józwik et al 2002 | 0.40 (0.24-0.55) |
| Combined | 0.37 (0.25-0.49) |
