# Supplementary figures and images for: Molecular and serological surveys of canine distemper virus: A meta-analysis of cross-sectional studies
Source: PLoS One. 2019 May 29;14(5):e0217594. doi: 10.1371/journal.pone.0217594 (PMC6541297; doi:10.1371/journal.pone.0217594)

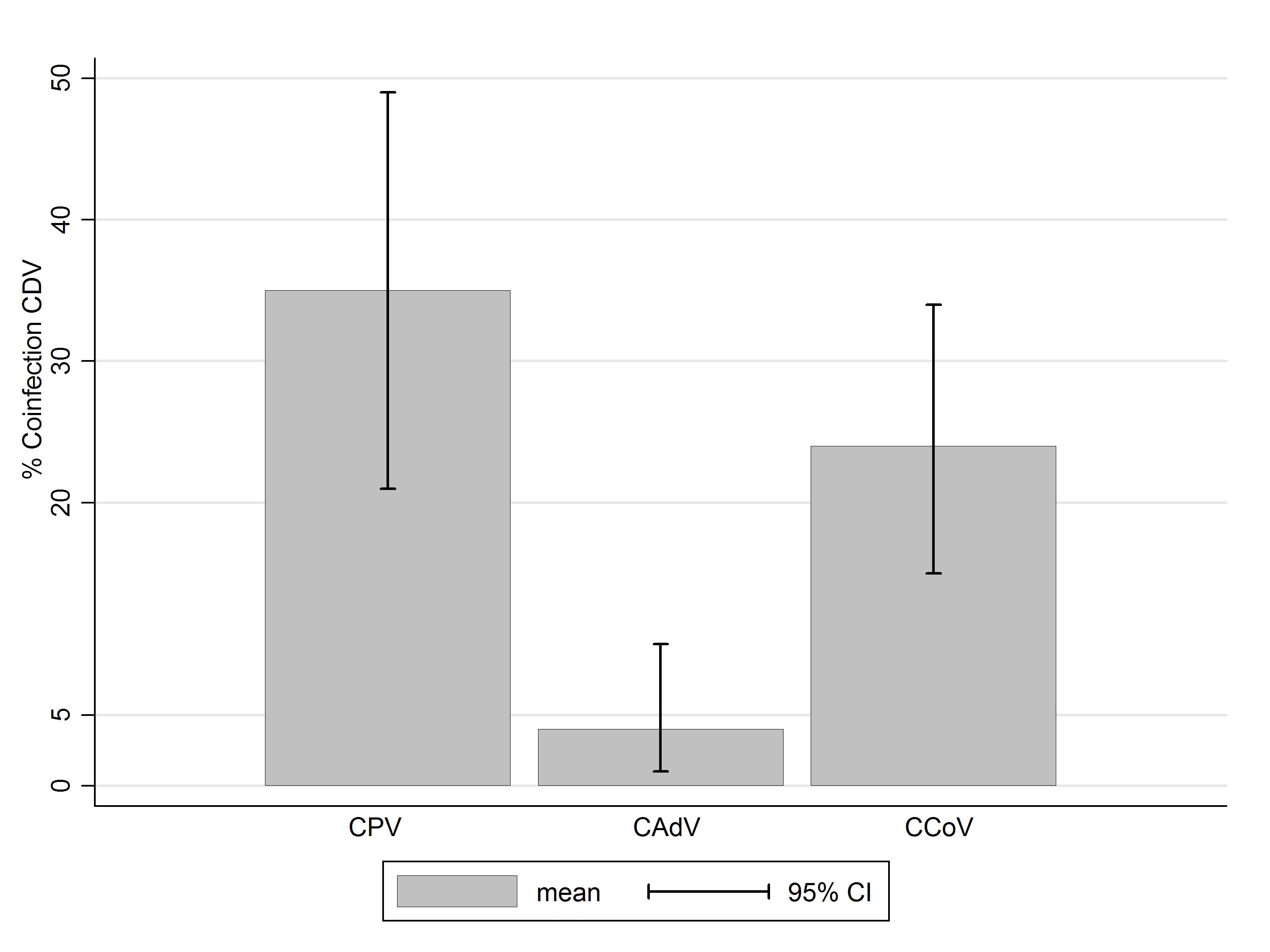

Supplement: S1 Fig — (TIF) [file pone.0217594.s008.tif]

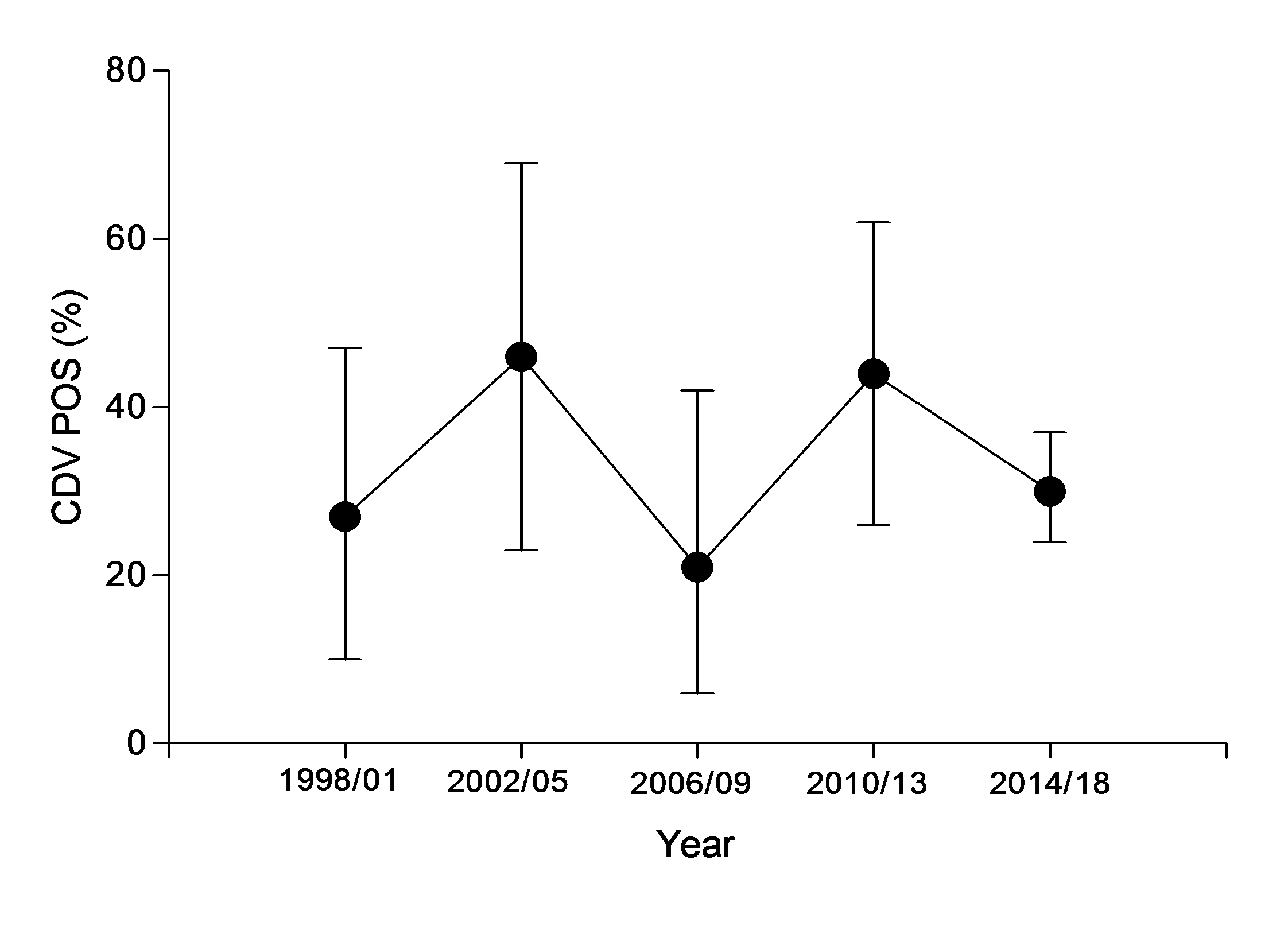

Supplement: S2 Fig — The variation corresponds to the 95% CI. (TIF) [file pone.0217594.s009.tif]

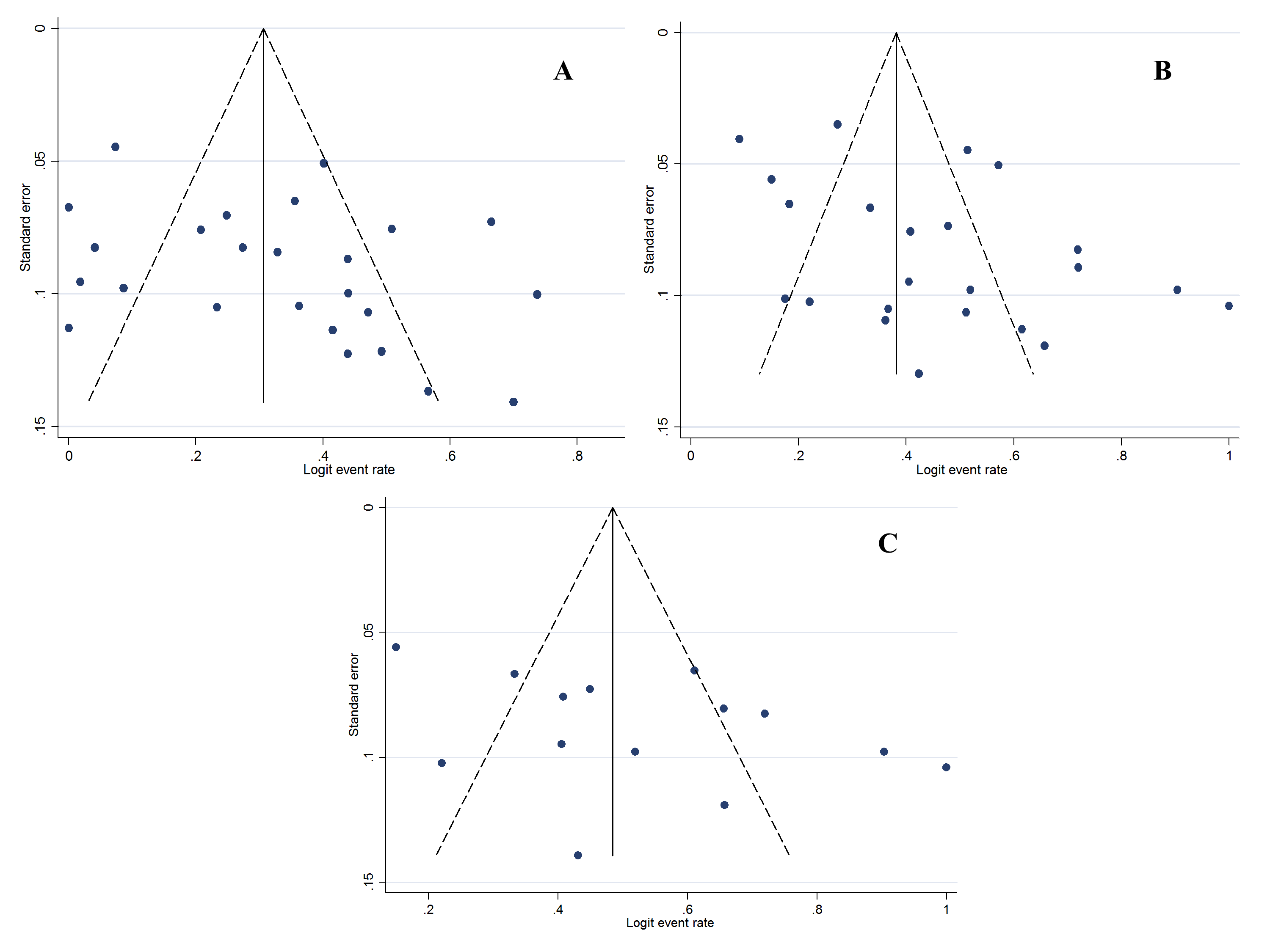

Supplement: S3 Fig — Funnel plot for CDV frequencies in subgroups molecular surveys (A), serological surveys (B) and free-ranging dogs (C). (TIF) [file pone.0217594.s010.tif]
